# Persistent viral control status is associated with enhanced innate immune responses in people with HIV-1

**DOI:** 10.1016/j.isci.2026.114807

**Published:** 2026-01-30

**Authors:** Jéssica C. dos Santos, Albert L. Groenendijk, Suzanne D.E. Ruijten, Rainer Knoll, Nadira Vadaq, Rob ter Horst, Ezio T. Fok, Wojciech Witkowski, Marc J.T. Blaauw, Louise E. van Eekeren, Wilhelm A.J. W. Vos, Maartje Cleophas-Jacobs, Stephan Reichl, Twan Otten, Joost H.A. Martens, Arnold van der Meer, Han Koninkx, Marien I. de Jonge, Marc D. Beyer, Jan van Lunzen, Leo A.B. Joosten, Christoph Bock, Casper Rokx, Annelies Verbon, Linos Vandekerckhove, Anna C. Aschenbrenner, Joachim L. Schultze, Vasiliki Matzaraki, Andre J.A.M. van der Ven, Mihai G. Netea

**Affiliations:** 1 Department of Internal Medicine and Infectious Diseases, Radboud University Medical Center, Nijmegen, the Netherlands; 2 Department of Internal Medicine and Department of Medical Microbiology and Infectious Diseases, ErasmusMC, Erasmus University, Rotterdam, the Netherlands; 3 Systems Medicine, Deutsches Zentrum für Neurodegenerative Erkrankungen (DZNE) eingetragener Verein (e.V.), Bonn, Germany; 4 Genomics & Immunoregulation, Life & Medical Sciences (LIMES) Institute, University of Bonn, Bonn, Germany; 5 Center for Molecular Medicine (CeMM) Research Center for Molecular Medicine of the Austrian Academy of Sciences, Vienna, Austria; 6 Medical University of Vienna, Center for Medical Statistics, Informatics and Intelligent Systems (CeMSIIS), Institute of Artificial Intelligence, Vienna, Austria; 7 Department of Molecular Biology, Radboud University, Nijmegen, the Netherlands; 8 HIV Cure Research Center, Department of Internal Medicine and Pediatrics, Ghent University Hospital, Ghent University, Ghent, Belgium; 9 Department of Laboratory Medicine, Laboratory of Medical Immunology, Radboud Medical Center, Nijmegen, the Netherlands; 10 Immunogenomics & Neurodegeneration, Deutsches Zentrum für Neurodegenerative Erkrankungen (DZNE) (e.V.), Bonn, Germany; 11 PRECISE Platform for Single Cell Genomics and Epigenomics, DZNE and University of Bonn and West German Genome Center, Bonn, Germany; 12 Department of Medical Genetics, Iuliu Hatieganu University of Medicine and Pharmacy, Cluj-Napoca, Romania; 13 Department of Immunology and Metabolism, Life and Medical Sciences Institute, University of Bonn, Bonn, Germany

**Keywords:** Health sciences, Medicine, Immunology, Clinical finding, Disease

## Abstract

The mechanisms mediating elite and persistent HIV control in people living with HIV (PLHIV) are only partially understood and largely attributed to adaptive T cell responses, but whether innate immunity also contributes remains unclear. Using samples from the 2000HIV study, we examined the transcriptional and functional profiles of monocytes from spontaneous HIV controllers and normal progressors on long-term antiretroviral therapy. HIV controllers displayed enhanced cytokine production after bacterial and viral stimulation, alongside antiviral and interferon-inducible transcriptional signatures and reduced inflammatory gene expression. Persistent controllers further showed increased capacity for trained immunity, with H3K4me3 profiling indicating the epigenetic priming of innate immune genes. Remarkably, relatives of persistent controllers also exhibited stronger innate and trained immune responses than relatives of normal progressors. These findings suggest that robust innate immunity, particularly monocyte function, may precede infection and contribute to sustained HIV control, offering new avenues for therapies that induce similar innate antiviral responses.

## Highlights

• Monocytes from persistent HIV controllers show enhanced cytokine responses upon stimulation
• Elite controllers monocytes show antiviral signatures with increased IFN-inducible genes
• Persistent controllers exhibit trained immunity with H3K4me3 priming of innate immune genes
• Relatives of persistent HIV controllers show stronger innate and trained immunity responses

## Graphical abstract

**Figure:**
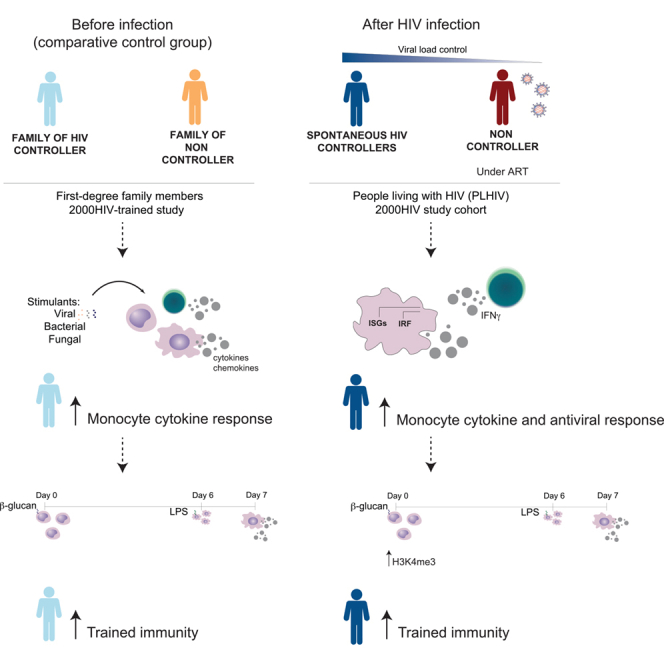

## Introduction

HIV controllers are a small minority of people living with HIV (PLHIV),^1^ who durably control HIV replication to undetectable or very low plasma viral load levels without anti-retroviral therapy (ART). HIV controllers are classified as either elite controllers (ECs) or viremic controllers (VCs), depending on plasma viral loads and CD4^+^ T cell counts.^2^ About 30% of HIV controllers, named as transient controllers, lose control over time. Although emerging evidence has identified potential contributing factors, the biological mechanisms driving this loss of control are not yet fully elucidated.^3^^,^^4^^,^^5^ Genetic variants in class I human leukocyte antigen (HLA) and the chemokine receptor CCR5 regions are one of the factors playing a role in HIV control.^6^^,^^7^ Apart from genetic traits, ECs exhibit distinctive HIV reservoir characteristics, with transcriptionally active proviruses preferentially located in centromeric satellite DNA or in zinc finger genes, both accompanied by heterochromatin features, while defective provirus sequences are found in permissive euchromatin regions.^8^^,^^9^^,^^10^ However, the mechanism underlying this unique HIV reservoir configuration in ECs remains unknown. Currently, the maintenance of functional HIV-specific CD8^+^ T cells is believed to be the main link between the protective immune responses and spontaneous HIV control.^11^^,^^12^

Evidence on the role of innate immune responses in HIV control has recently emerged. Myeloid dendritic cells (DCs) show augmented response, associated with a unique metabolic profile in ECs,^13^^,^^14^ while natural killer (NK) cells display enhanced cytotoxicity and survival.^14^^,^^15^ ECs have higher circulating concentrations of the chemokines CCL14, CCL21, CCL27, and CXCL1, known to suppress HIV replication in resting CD4^+^ T cells.^16^ Studies assessing the contribution of other myeloid cells, such as monocytes, to the HIV controller status reported the presence of monocytes with the increased expression of the IFN-inducible genes *IFIT1* and *IFIT3*. In addition, increased IL-1β production in response to LPS was observed in monocytes of elite controllers compared to uninfected controls and ART recipients.^17^ In addition, Tilton and colleagues showed that increased viremia associates with the diminished production of inflammatory cytokines by monocytes.^18^ However, a comprehensive understanding of the functional programs in innate immune cells of HIV controllers is missing, and whether this is an intrinsic characteristic of these individuals before they are infected by HIV is unknown.

In the present study, we assessed whether monocyte responses contribute to both the establishment and maintenance of HIV control status. To investigate this hypothesis, we studied monocyte functions from HIV controllers and normal progressors on long-term ART, as well as in their first-degree relatives as surrogates for their immune responses before HIV infection. Our findings underscore the significant contributions of monocytes to initiate and sustain HIV control, and we propose a trained immunity phenotype as a potential mechanism that underpins the resilience of the innate immune responses in ECs.

## Results

### Monocyte responses of persistent HIV controllers are increased upon stimulation

An extensive description of the cohort, including normal progressor (non-controllers) and the sub-phenotypes of various HIV controllers, including elite controllers (ECs), viremic controllers (VCs), and transient controllers (TCs), is presented in Table 1. TCs are PLHIV who exhibited plasma HIV-RNA levels exceeding 10,000 copies/mL after initially meeting the criteria for being classified as an HIV controller. In contrast, persistent HIV controllers are individuals who managed to maintain their HIV-RNA levels less than 10,000 copies/mL. Specifically, as part of the persistent HIV controllers group, persistent ECs are individuals who managed to maintain their HIV-RNA levels less than 75 copies/mL during the entire ART-naive follow-up period.

**Table 1:** Characteristics of 2000HIV-study participants

	DISCOVERY			VALIDATION		
	HIV controllers (*n* = 102)	Non-HIV controllers (*n* = 1407)	*p*-*value*^c^	HIV controllers (*n* = 12)	Non-HIV controllers (*n* = 314)	*p*-*value*^c^
**HIV Controllers definition, number (%)**						

Elite controllers on ART	3 (2.9%)	0 (0%)	–	0 (0%)	0 (0%)	–
Elite controllers	21 (20.6%)	0 (0%)	–	0 (0%)	0 (0%)	–
Viremic controllers on ART	28 (27.5%)	0 (0%)	–	3 (25.0%)	0 (0%)	–
Viremic controllers	5 (4.9%)	0 (0%)	–	0 (0%)	0 (0%)	–
Transient controllers on ART	45 (44.1%)	0 (0%)	–	9 (75.0%)	0 (0%)	–

**Sex**						

Male	76 (74.5%)	1207 (85.8%)	0.003	8 (66.7%)	266 (84.7%)	ns
Female	26 (25.5%)	200 (14.2%)	–	4 (33.3%)	48 (15.3%)	–

**Age (years)**						

Mean (SD)	51.0 (11.8)	51.5 (11.8)	–	52.3 (5.97)	53.1 (11.1)	–
Median [Min, Max]	51.5 [28.0, 77.0]	53.0 [19.0, 84.0]	ns	51.0 [41.0, 64.0]	53.0 [20.0, 77.0]	ns

**Ancestry, number (%)**						

Asian	5 (4.9%)	69 (4.9%)	0.0012	0 (0%)	11 (3.5%)	ns
African	13 (12.7%)	149 (10.6%)	–	2 (16.7%)	22 (7.0%)	–
Hispanic	3 (2.9%)	42 (3.0%)	–	0 (0%)	2 (0.6%)	–
Mixed ancestry	19 (18.6%)	102 (7.2%)	–	0 (0%)	6 (1.9%)	–
Native American	0 (0%)	0 (0%)	–	0 (0%)	2 (0.6%)	–
European	62 (60.8%)	1043 (74.1%)	–	10 (83.3%)	271 (86.3%)	–

**VL latest, copies/mL**						

Undetectable^d^	93 (91.2%)	1369 (97.3%)	0.00167	11 (91.7%)	303 (96.5%)	ns
Detectable^e^	9 (8.8%)	38 (2.7%)	–	1 (8.3%)	11 (3.5%)	–

**VL latest, (for detectable means)**						

Mean (SD^a^)	316 (390)	64.3 (59.9)	–	99.0 (NA)	48.4 (28.3)	ns
Median [Min, Max^b^]	166 [27.0, 1110]	43.5 [21.0, 400]	0.0117	99.0 [99.0, 99.0]	37.0 [21.0, 121]	–

**CD4 nadir, cells/μL**						

Median [Min, Max^b^]	450 [470, 2210]	240 [0, 1410]	<2.2 × 10^−16^	355 [180, 690]	260 [0, 920]	0.03771
Missing	0 (0%)	26 (1.8%)	–	0 (0%)	9 (2.9%)	–

**CD4 latest, cells/μL**						

Median [Min, Max]	753 [350, 3170]	709 [66, 2200]	0.00787	665 [390, 1130]	660 [120, 1660]	ns
Missing	0 (0%)	15 (1.1%)	–	0 (0%)	0 (0%)	–

**HIV duration, years**						

Mean (SD)	16.5 (7.73)	14.1 (8.20)	–	16.3 (6.08)	11.6 (7.92)	–
Median [Min, Max]	15.1 [1.49, 39.0]	13.0 [0.500, 42.0]	0.00168	17.7 [5.31, 24.0]	10.2 [0.510, 37.0]	0.02058

**Season of inclusion, numbers**						

spring	23 (22.5%)	212 (15.1%)	0.00058	0 (0%)	93 (29.6%)	0.017
summer	45 (44.1%)	466 (33.1%)	–	8 (66.7%)	90 (28.7%)	–
autumn	24 (23.5%)	356 (25.3%)	–	0 (0%)	27 (8.6%)	–
winter	10 (9.8%)	373 (26.5%)	–	4 (33.3%)	104 (33.1%)	–

**CMV Serostatus**						

CMV (−)	8 (7.8%)	79 (5.6%)	ns	2 (16.7%)	26 (8.3%)	ns
CMV (+)	94 (92.2%)	1323 (94.0%)	–	10 (83.3%)	286 (91.1%)	–
Missing	0 (0%)	5 (0.4%)	–	0 (0%)	2 (0.6%)	–

a SD, standard deviation.

b Min, Max, Minimum - maximum.

c *p-value*, comparison between HIV controllers and non-HIV controllers. Statistics: Fisher’s exact and Mann-Whitney U tests for categorical and continuous variables, respectively.

d Unmeasurable, unquantifiable or <40 copies/mL.

e >40 copies/mL, exact quantification.

**Figure 1:**
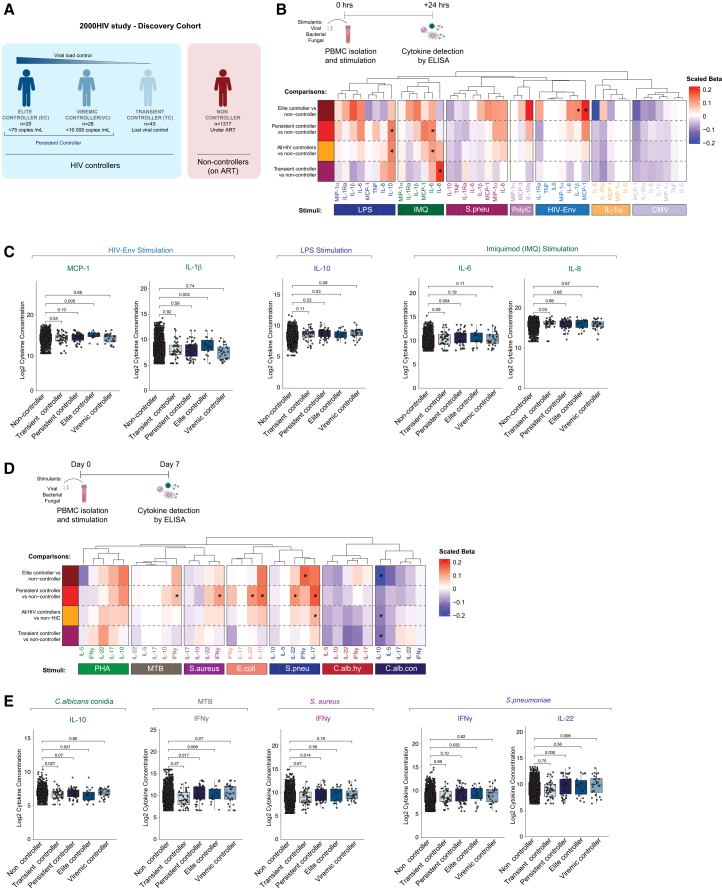
Monocytes and lymphocytes' responses to stimulation (A and B) PBMCs from HIV controllers (*n* = 95), consisting of elite controllers (ECs, *n* = 20), viremic controllers (VCs, *n* = 28) [persistent HIV controllers], transient controllers (TCs, *n* = 43) and normal progressor on ART (*n* = 1317) were stimulated with the TLR ligands Poly:IC, IMQ, LPS, the peptides HIV ENV pool and pp65 CMV, *S. pneumoniae* and IL-1α for 24 h and cytokines (IL-10, IL-1β, IL1-Ra, IL-6, IL-8, TNF) and chemokines (MCP-1, MIP-1α) were measured by ELISA. (C) Log2-transformed concentration of MCP-1, IL-1β, IL-10, IL-6, and IL-8 upon stimulation across the different groups of PLHIV. (D) PBMCs from the same groups of individuals were stimulated for 7 days with *C. albicans* conidia, *C. albicans* hyphae, *E. coli*, *M. tuberculosis,* PHA, *S. aureus*, *S. pneumoniae,* and cytokines (IFNγ, IL-10, IL-17, IL-22, and IL-5) were measured by ELISA. (E) Log2-transformed concentration of IL-10, IFNγ, and IL-22 upon stimulation across the different groups of PLHIV. Rank-based regression with age, sex, seasonality, and ancestry as confounders was used for *p*-value calculation, ∗*nominal p <* 0.05. Heatmaps represent the estimate from rfit divided by the mean cytokine level (scaled beta-values). The data are represented in boxplots showing the median (horizontal line) with standard errors.

Production of cytokines was assessed in PBMCs from ECs (*n* = 20), VCs (*n* = 28), TCs (*n* = 43), and non-controllers (*n* = 1317) after stimulation with bacterial (heat-killed *Streptococcus pneumoniae*), fungal (heat-killed *Candida albicans*), viral (HIV envelope (HIV-Env) and pp65-cytomegalovirus (CMV) peptide pools), and Toll-like receptor (TLR) ligands (imiquimod(IMQ), lipopolysaccharide (LPS), and Poly:IC) (Figures 1A and 1B). PBMCs from all HIV controllers produced more IL-6 and IL-10 upon 24 h stimulation with IMQ (TLR7 agonist) and LPS (TLR4 agonist), respectively, compared to non-controllers. This increase in IL-6 and IL-10 production remained when persistent controllers (ECs and VCs) were compared with non-controllers (Figures 1B and 1C, nominal *p* < 0.05). IL-1β and MCP-1 (CCL2) production was higher after the stimulation of PBMCs of ECs with HIV-Env peptide pool compared to non-controllers (Figures 1B and 1C, nominal *p* < 0.05). IL-8 production was higher in cells of transient controllers vs. non-controllers upon IMQ exposure (Figures 1B and 1C, nominal *p* < 0.05). After 7-day stimulation needed for the production of lymphocyte-derived cytokines (Figure 1D), PBMCs from persistent controllers showed increased production of IFNγ, IL-22, IL-10, and IL-17 upon stimulation with various stimuli (Figures 1D and 1Enominal *p* < 0.05). ECs showed a lower IL-10 and higher IFNγ production than non-controllers after stimulation with *C. albicans* conidia and *S. pneumoniae*, respectively (Figures 1D and 1E, nominal *p* < 0.05). Of note, no significant differences were observed in the absolute numbers of monocytes and lymphocytes within different groups of HIV controllers compared to non-controllers (Figure S1A). This indicates that the differences in immune responses upon stimulation are the result of an enhancement in the function of the immune cells of HIV controllers rather than an increase in the number of cells (Figure S1A). Collectively, we observed stronger monocyte-derived immune responses as well as higher production of lymphocyte-derived cytokines such as IFNγ in HIV controllers compared to non-controllers after the stimulation of PBMCs.

### Monocytes transcriptional signatures of elite controllers show enhanced expression of interferon-inducible genes and a decrease in inflammation-related gene expression

**Figure 2:**
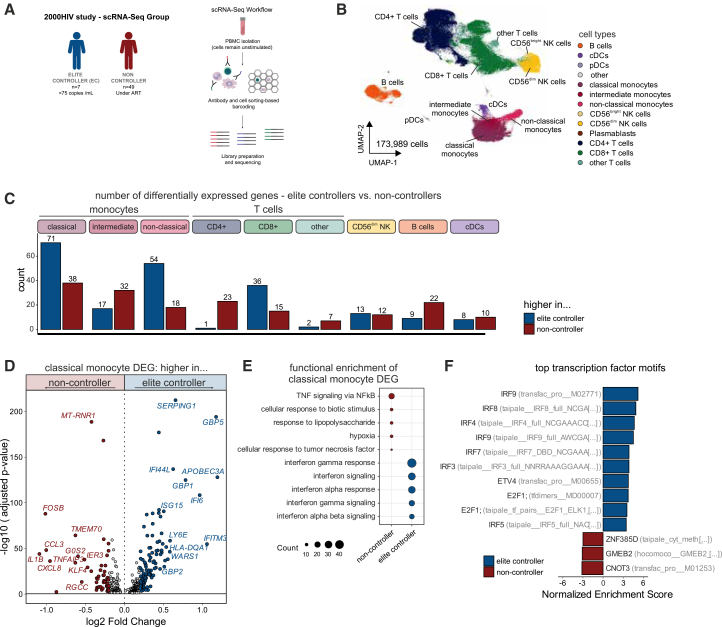
Monocyte transcriptome at single-cell level (A) Single-cell (sc) RNA-sequencing data were generated from PBMCs of ECs (*n* = 7) and non-controllers (*n* = 49) matched by age, sex, and seasonality were analyzed. For robustness, permutations (*n* = 1000) of equal group sizes were performed. (B) UMAP of PBMCs from all groups, indicating the identified cell types. (C) Number of DEG by the major cell types after comparison between ECs and non-controllers. (D) DEG by classical monocytes comparing ECs with non-controllers. (E) Functional enrichment using GO, Reactome, and Hallmark databases for ECs-specific up- and down-regulated genes in classical monocytes. (F) Normalized enrichment score (NES) of the top TFs motif enriched in classical monocytes of ECs and non-controllers.

We previously demonstrated that PLHIV on stable ART exhibited sustained systemic inflammation with enhanced IL-1β transcription, compared to healthy controls.^19^^,^^20^ To determine molecular phenotypes and transcriptional differences between HIV controllers and non-controllers that could explain the different immune response to stimuli, we generated transcriptome data at the single-cell (sc) level of PBMCs from a subgroup of age-, sex- and season-matched HIV elite controllers (*n* = 7) as well as non-controllers (*n* = 49) (Figure 2A). The resulting data comprised 173,989 single-cell transcriptomes, and all expected cell types were identified (Figures 2B and S1B). The assessment of differentially expressed genes (DEGs) between ECs and non-controllers across all immune populations demonstrated classical monocytes presenting the highest number of DEGs (log_2_FC ≥ 0.25, adjusted *p*-value < 0.05) (Figure 2C). Inspecting the DEGs for ECs against non-controllers in classical monocytes in more detail showed that ECs were strongly enriched with interferon-induced genes such as *GBP5, GBP1, GBP2, APOBEC3A, ISG15, IFI6, IFI44L,* and *IFITM3*, while non-controllers had higher expression of pro-inflammatory genes, including *FOSB, IL1B, CCL3* (MIP-1α)*, CXCL8*, encoding for IL-8 (Figure 2D). The robustness of these DEGs was tested by assessing their significance through 1,000 permutations. In each permutation, the EC group was held constant while an equally sized group of non-HIC samples was randomly selected for comparison (Figure S1C). Pathway analysis of monocyte DEGs reflected these findings with enrichment in pathways related to interferon signaling in ECs, while non-controllers showed enrichment in inflammatory pathways related to TNF (Figure 2E). To test if these changes are mainly observed in monocytes, we performed functional enrichment analysis using all major cell types (Figure S1D). EC-related interferon terms were also found in non-classical monocytes, as well as cDCs, B cells, and CD8^+^ T cells, while TNF signaling in non-controllers was also present in other cell types such as CD4^+^ T cells, albeit monocytes showed the strongest enrichment. DEGs for all major cell types are described in Table S1.

Next, to identify regulatory patterns, we assessed the landscape of transcription factors (TFs) associated with the DEG of ECs monocytes using TF motif analysis. In agreement with the gene expression profile, classical monocytes of ECs were enriched for genes regulated by TFs of the interferon-response, such as IRF9, IRF8, IRF4, IRF7, and IRF3 (Figure 2F). The exposure of monocytes to IFNs, both type I and II, stimulates the expression of IFN-stimulated genes (ISGs) through the phosphorylation of IRFs and STAT TFs.^21^^,^^22^ Given that the myeloid compartment in ECs showed clear evidence for interferon response signatures and interferon-related TF activity, we assessed IFNγ concentrations in plasma and identified increased concentrations in ECs compared to non-controllers (Figure S1E, nominal *p* < 0.05).

To determine whether the transcriptional differences result from changes in all classical monocytes or whether there are monocyte subgroups with specific transcriptional states, we subclustered the classical monocyte compartment, revealing four distinct states (Figures S1F and S1G). No significant differences were observed; however, a tendency of more monocytes with an inflammatory profile was observed for non-controllers (Figure S1H). In addition, lower sCD14 plasma concentrations, known to be shed from the membrane of monocytes, were observed in ECs compared to non-controllers (Figure S1I).

Collectively, these findings show that monocytes of ECs are transcriptionally characterized by an antiviral and interferon-related signature, whereas monocytes from non-controllers are enriched for genes involved in inflammatory processes.

### Genetic determinants of HIV control and cytokine responses in HIV controllers

**Figure 3:**
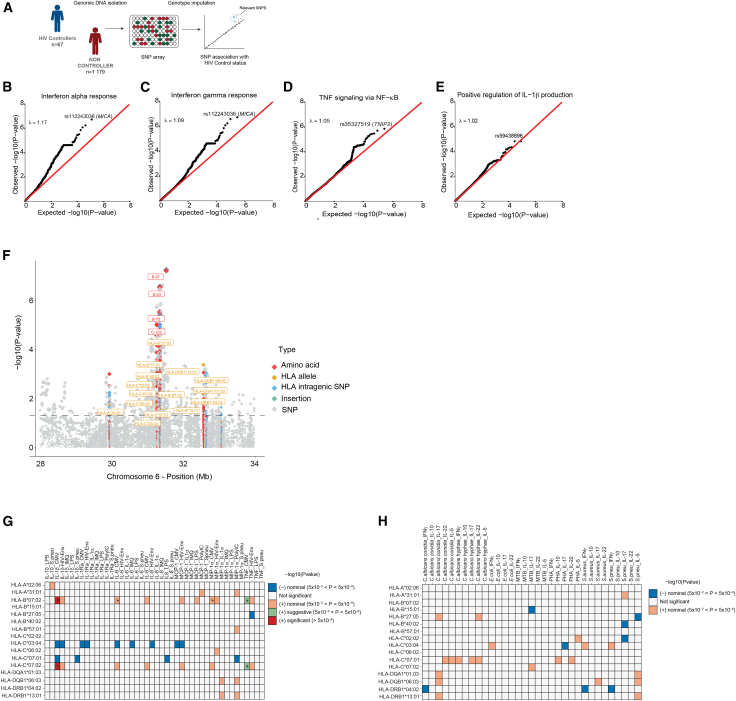
Association of genetic and HLA variants with HIV control and *ex vivo* cytokine production (A) Case-control genome-wide association study (GWAS) was performed in 67 HIV controllers and 1179 non-controllers of European ancestry. (B–E) Quantile-quantile (QQ)-plots of SNPs associated with HIV control in proximity to genes of signaling immune pathways, comparing expected GWAS -log_10_(*p*-values) (x axis) with observed GWAS -log_10_(*p*-values) (y axis). The dots show deviation from the expected line (λ_inflation_ > 1). SNPs were extracted within a 150 kb window from all genes in the pathways interferon alpha responses, interferon gamma responses, TNF signaling via NF-κB, and interleukin-1β production. (F) Manhattan plot of association results between SNPs (gray), classical HLA alleles (yellow), and amino acids (red) within the MHC region of Europeans HIV controllers (*n* = 67) and non-controllers (*n* = 1179). Association was tested using a firth logistic regression. X axis shows the physical position within the MHC region, and y axis the strength of associations (-log10(*p*-value)). Coordinates are based on GRCh37. The dotted line indicates the *P* nominal value 0.05). (G and H) Heatmaps showing the results of the association between HLA alleles associated with HIV control and *ex vivo* cytokine production upon 24-h and 7-day stimulation using the European discovery cohort. Different colors represent different thresholds of unadjusted *p* values as shown in the legend (−log10(*p-*value)). Asterisks indicate the associations that reached statistical significance after correction of the *p* value for multiple testing (*p* <1.13 × 10^−4^ and *p* <8.45 × 10^−5^ for 24-h and 7-day stimulation, respectively).

Single nucleotide polymorphisms (SNPs) and alleles in the in the major histocompatibility complex (MHC) region were previously identified as major genetic contributors for HIV control.^6^ To investigate if genetic predispositions could explain the observed differences in monocyte responses in ECs versus non-controllers, we first conducted a case-control genome-wide association study (GWAS) in 67 HIV controllers and 1179 non-controllers, respectively, to identify SNPs associated to HIV control (Figure 3A). Since population stratification is known to be an important confounder in genetic studies, we focused on individuals of European ancestry (Figure S2A). To increase the statistical power of the GWAS, persistent HIV controllers and TCs of Europeans ancestry are included as HIV controllers, as the short-term ability of TCs to achieve spontaneous HIV control may be influenced by genetic factors.

GWAS identified 19 independent and suggestive (*p* < 1 × 10^−5^, r^2^ < 0.1) SNPs associated with HIV control (Table S2). The strongest association with viral control was observed on chromosome 6, rs112243036 (*p =* 1.82 × 10^−7^; OR = 3.28), located in the MHC region (Figure S2B). The identified SNP, in close proximity to *HLA-B*, was in strong linkage disequilibrium (r^2^ 0.81) with rs4418214, previously identified as associated with HIV control in the International HIV Controllers Study (*p =* 1.4 × 10^−34^).^6^

Next, we evaluated SNPs in the vicinity ( ±150 kb window) of genes that are involved in the pathways found to be strongly modulated in monocytes of ECs in our single-cell transcriptomic analysis to determine whether these genetic variants were enriched in SNPs associated with HIV control. We observed an enrichment of SNPs associated with HIV control within genes of “interferon alpha and gamma responses” (λ_inflation_ = 1.17 and 1.09, respectively) (Figures 3B and 3C). The top enriched SNP was the strongest associated SNP with HIV control within the MHC region, rs112243036 (*p =* 1.82 × 10^−7^; OR = 3.28) (Figure S2B). In addition, a less pronounced enrichment of SNPs associated with HIV control was identified within genes of the “TNF signaling via NF-κB” pathway (λ_inflation_ = 1.05) (Figure 3D), followed by SNPs in genes of “IL-1β production” (Figure 3E). The top enriched SNP within genes of the “TNF signaling via NF-κB” pathway was rs35327519 (case-control GWAS *p* = 1.41 × 10^−6^; OR = 3.49), located near *TNIP2.* As for the “IL-1β production” pathway, the identified SNP rs59438896 (Case-control GWAS *p* = 1.50 × 10^−5^; OR = 3.29) was located near *MICA/B* genes.

Given the extensive linkage disequilibrium (LD) in the MHC region, to achieve better resolution, we next imputed classical HLA alleles at four-digit resolution and amino acid polymorphisms in the same group of European HIV controllers and non-controllers subjects as used in the GWAS. We first tested the concordance of MHC imputation by generating HLA typing data for 104 samples using next-generation sequencing (NGS). The concordance between the imputed and NGS type data at four-digit resolution was 99% for most of the alleles, with a few exceptions of class II alleles presenting concordance below 85% (Figure S2C). Next, we tested which HLA alleles and amino acids were associated with HIV control through firth logistic regression, adjusting for the same covariates (age, sex, and first 5 genetic PCs) used in the GWAS [see STAR Methods]. HLA *B∗27:05* showed the strongest association (OR = 3.72, *p* = 2.64 × 10^−5^) followed by *B∗57:01* (OR = 3.57, *p* = 2.07 × 10^−4^). The strongest associations of amino acids (*p* < 1 × 10^−5^) were identified at position 70 within HLA-B (*P*_omnibus_ = 2.31 × 10^−6^) and position 152 (*P*_omnibus_ = 8.97 × 10^−6^) within HLA-C (Table S1).

HLA alleles *B∗27:05* and *B∗57:01* and amino acids within HLA-B, including amino acid 70, were previously identified as associated with HIV control^6^ (Figure 3F; Tables 2 and S3). As the differences in HLA alleles are involved in antigen-presentation processes and may indirectly modulate the innate immune responses of monocytes upon microbial exposure through intermediary IFN-gamma release, we next investigated whether HLA alleles associated with HIV control influence cytokine production in HIV controllers. For this, we tested the association between the HLA alleles associated with HIV control at a *P* nominal significance (*p* < 0.05, *n* = 16) and cytokine production upon 24-h stimulation with various stimuli using individuals of European ancestry from the discovery cohort (Figure 3G) and validation cohort. Associations that showed a *p* value after multiple testing <1.13 × 10^−4^ in the discovery cohort and a nominal *p* < 0.05 in the validation cohort were considered statistically significant (Table S2). In general, we observed a limited impact of genetic variants in HLA on proinflammatory cytokine production. No significant associations were observed for *B∗27:05* and *B∗57:01* with monocyte-derived cytokines upon stimulation. However, we observed that *B∗07:02* and *C∗07:02* alleles showed a statistically significant positive association with IL-1β and TNF production only upon CMV stimulation (beta 0.54 and 0.45 for IL-1β and TNF, respectively). Notably, both *B∗07:02* (OR = 0.25, *P* 3.55 × 10^−3^) and *C∗07:02* (OR = 0.28, *P* 3.0 × 10^−3^) were associated with a reduced likelihood of being an HIV controller in our cohort (Table 2). In addition, *B∗07:02* significantly impacted the IL-6 and MIP-1α production upon CMV stimulation in the discovery cohort (*p* value after multiple testing <1.13 × 10^−4^) (Figure 3G), but not in the validation cohort (*p* < 0.05). In addition to monocyte-derived responses, we also tested the association between the HLA alleles associated with HIV control and lymphocyte-derived cytokine production upon 7-day stimulation in Europeans from the discovery cohort (Figure 3H) and validation cohort. The HLA alleles associated with HIV control did not show a statistically significant association after multiple testing correction, as shown in Figure 3H.

**Table 2:** HLA alleles associated with HIV control in Europeans of the 2000HIV cohort

Chr^a^	HLA type	Position	OR^b^	*p-*value^c^
6	HLA-B∗27:05	31321693	3.727	2.64 × 10^−5^
6	HLA-B∗57:01	31321788	3.578	2.07 × 10^−4^
6	HLA-DRB1∗13:01	32546596	2.547	4.19 × 10^−4^
6	HLA-DQB1∗06:03	32627269	2.386	9.08 × 10^−4^
6	HLA-B∗40:02	31321727	3.594	1.49 × 10^−3^
6	HLA-C∗02:02	31236530	2.553	2.10 × 10^−3^
6	HLA-DQA1∗01:03	32605186	2.224	2.40 × 10^−3^
6	HLA-C∗07:02	31236553	0.2847	3.00 × 10^−3^
6	HLA-B∗07:02	31321650	0.2599	3.55 × 10^−3^
6	HLA-DRB1∗04:02	32546557	6.534	6.93 × 10^−3^
6	HLA-C∗06:02	31236550	1.933	1.31 × 10^−2^
6	HLA-C∗03:04	31236536	1.875	1.76 × 10^−2^
6	HLA-A∗31:01	29910315	2.545	1.80 × 10^−2^
6	HLA-C∗07:01	31236552	0.5159	2.23 × 10^−2^
6	HLA-B∗15:01	31321664	1.844	2.83 × 10^−2^
6	HLA-A∗02:06	29910257	5.54	3.73 × 10^−2^

a Chr, chromosome.

b OR, odds ratio.

c *p-value*, logistic regression model adjusted for age, sex, and the first five genetic PCs as covariates.

### Monocytes of elite controllers display an increased *trained immunity* phenotype

**Figure 4:**
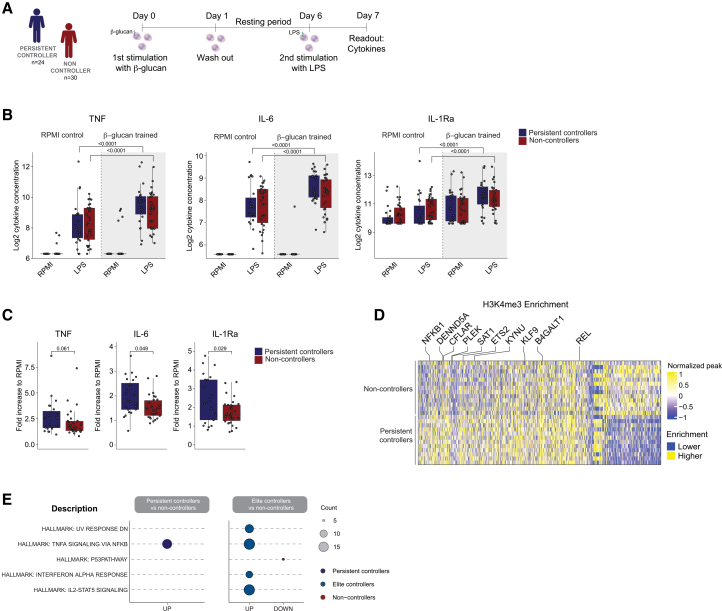
Induction of trained immunity in persistent controllers and non-controllers (A) Overview of the participants of the 2000HIV-trained study. Adherent monocytes from persistent HIV controllers (*n* = 24), of whom 16 were EC, and 8 were VC, and non-controllers (*n* = 30) were exposed to *C. albicans* β-glucan for 24 h and restimulated with LPS at day 6. (B) Cytokines (TNF, IL-6, and IL-1Ra) concentrations were measured by ELISA after the induction of trained immunity in the two groups of PLHIV. Comparisons between trained monocytes and RPMI control; Comparisons between persistent controllers vs. non-controllers before and after training. For determination of statistical differences, a Wilcoxon rank-sum test was performed (*p <* 0.05). The data are represented as boxplots with median and standard errors. (C) Fold change in TNF, IL-6, and IL-1Ra production upon β-glucan training normalized to RPMI. (D) Heatmap showing the normalized H3K4me3 deposition at promoters of CD14^+^ monocytes from persistent HIV controllers (*n* = 12) of whom 4 were ECs and non-controllers (*n* = 13). (E) Functional enrichment using Hallmark database for persistent controllers- and EC-specific up- and down-regulated regions in monocytes.

As genetic variants can partially explain the increased monocyte cytokine production in HIV controllers, we next evaluated whether non-genetic regulation of monocyte function may explain these effects. Trained immunity has been described as the immunological process induced by an initial stimulus that confers innate immune cells a long-term ability to mount more robust host responses upon exposure to secondary stimulations.^23^^,^^24^ We studied the induction of trained immunity by exposing monocytes to β-glucan, a well-known inducer of trained immunity, followed by restimulation with LPS, in 25 persistent controllers, 17 ECs and 8 VCs, and 30 non-controllers as part of the 2000HIV-trained study matched by age, sex, and ancestry (Figure 4A; Table 3). β-glucan training alone, without the restimulation of cells with a secondary stimulus, induced only the production of IL-1Ra, but not of TNF and IL-6. No differences in the direct induction of cytokine production were observed between persistent controllers and non-controllers (Figure 4B). In contrast, persistent controllers showed an increased trained immunity capacity measured by a higher fold increase of IL-6 and IL-1Ra production upon LPS restimulation, compared to non-controllers (Figure 4C; *p <* 0.05). We next evaluated the presence of H3K4me3 in promoters of immune genes, as a histone mark associated with a trained immunity phenotype.^25^ A higher number of regions enriched for H3K4me3 was observed in persistent controllers compared to non-controllers resting CD14^+^ monocytes (562 up- and 196 downregulated regions Figure 4D). The primed regions in HIV controllers were enriched in the pathways “TNF signaling via NF-κB,” “IL2 STAT5 signaling,” and “interferon alpha response,” whereas the downmodulated regions were enriched for the “P53 pathway” (Figure 4E; *p* < 0.05). Altogether, our results indicate that monocytes of HIV controllers present an increased capacity to mount effective trained immunity responses, which are associated with a modified epigenetic landscape.

**Table 3:** Characteristics of 2000HIV-trained sub-study participants

	HIV controllers (ECs and VCs) (*n* = 25)	Non-HIV controllers (*n* = 30)	*p*-value^c^	Family of HIV controllers (*n* = 19)	Family of non-HIV controllers (*n* = 21)	*p*-Value^d^
**Sex**						

Female	8 (32.0%)	7 (23.3%)	ns	14 (73.7%)	14 (66.7%)	ns
Male	17 (68.0%)	23 (76.7%)	–	5 (26.3%)	7 (33.3%)	–

**Age (years)**						

Mean (SD^a^)	50.6 (14.2)	53.8 (10.6)	–	52.3 (13.5)	54.1 (19.0)	ns
Median [Min, Max^b^]	49.0 [28.0, 78.0]	55.0 [31.0, 75.0]	ns	57.0 [29.0, 72.0]	57.0 [18.0, 85.0]	–

**Ancestry, number (%)**						

European	17 (68.0%)	24 (80.0%)	ns	15 (78.9%)	19 (90.5%)	ns
African	3 (12.0%)	2 (6.7%)	–	1 (5.3%)	0 (0%)	–
Asian	2 (8.0%)	1 (3.3%)	–	0 (0%)	0 (0%)	–
Hispanic	1 (4.0%)	1 (3.3%)	–	2 (10.5%)	1 (4.8%)	–
Other	2 (8.0%)	2 (6.7%)	–	1 (5.3%)	1 (4.8%)	–

**Family-PLHIV relationship**						

sibling	–	–	–	13 (68.4%)	12 (57.1%)	–
parent	–	–	–	6 (31.6%)	6 (28.6%)	–
child	–	–	–	0 (0%)	3 (14.3%)	–

a SD, standard deviation.

b Min, Max, Minimum - maximum.

c *p-value*, comparison between HIV controllers and non-HIV controllers.

d *p-value*, comparison between relatives of HIV controllers and relatives of non-HIV controllers. Statistics: Fisher’s exact and Mann-Whitney U tests for categorical and continuous variables, respectively.

### Increased innate immune responses in first-degree family members of HIV controllers

**Figure 5:**
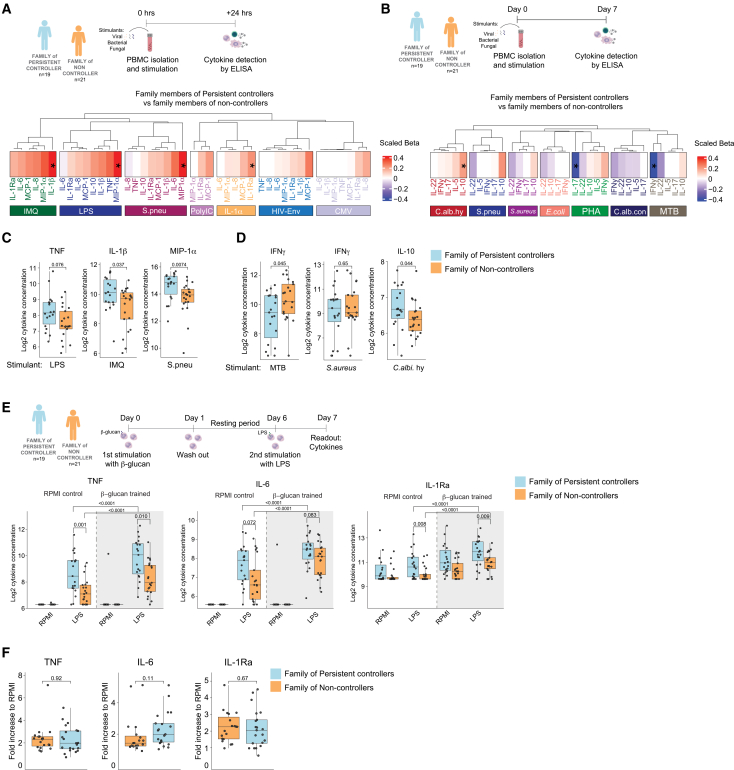
Functional examination of monocytes and lymphocytes in first-degree family members of persistent controllers and non-controllers (A) PBMCs from first-degree family members of persistent controllers (*n* = 19) and non-controllers (*n* = 21) matched by age, sex, and ancestry were stimulated with the TLR ligands Poly:IC, IMQ, LPS, the peptides HIV ENV pool and pp65 CMV, *S. pneumoniae,* and rhIL-1α for 24 h, and cytokines (IL-10, IL-1β, IL1-Ra, IL-6, IL-8, TNF) and chemokines (MCP-1, MIP-1α) were measured by ELISA. (B) PBMCs from the same group of individuals were stimulated for 7 days with *C. albicans* conidia, *C. albicans* hyphae, *E. coli*, *M. tuberculosis,* PHA, *S. aureus*, *S. pneumoniae,* and the cytokines (IFNγ, IL-10, IL-17, IL-22, IL-5) were measured by ELISA. Heatmaps represent the estimate divided by the mean cytokine level (scaled concentration). Statistical significance was assessed by the Wilcoxon rank-sum test. (C) Log2 TNF, IL-1β, and MIP-1α concentration upon stimulation across the different groups of family members. (D) Log2 IFNγ and IL-10 concentration upon stimulation across the different groups of family members. (E) Adherent monocytes from the family of persistent controllers and family of non-controllers were exposed to *C. albicans* β-glucan for 24 h and restimulated with LPS at day 7. Cytokines (TNF, IL-6, and IL-1Ra) concentrations were measured by ELISA. (F) Fold increase in TNF, IL-6, and IL-1Ra production upon β-glucan training normalized to RPMI. Comparisons between trained monocytes and RPMI control; Comparisons between family members of persistent controllers vs. family of non-controllers, before and after training. For the determination of statistical significance Wilcoxon rank-sum test was performed (*p <* 0.05). The data are represented as boxplots showing the median with standard errors.

As studies in PLHIV do not allow the distinction between an immune phenotype already present before HIV infection and one induced by the viral infection itself during the early stages of the infection, we investigated the immune responses of uninfected first-degree family members of persistent viral controllers (*n* = 19) and normal progressors (*n* = 21) matched by age, sex and ancestry (Table 3). The immune system of family members can potentially mirror the immune systems of PLHIV prior to infection. Similarly to the 2000-HIV study, we evaluated both monocyte- and lymphocyte-derived cytokines and chemokines upon stimulation (Figures 5A and 5B). Monocytes from family members of persistent controllers showed increased production of TNF, MIP-1α (CCL3) and IL-1β after exposure to LPS, *S. pneumoniae* and IMQ compared to those from family members of non-controllers monocytes (Figures 5A and 5C, nominal *p* < 0.05). Differences in the production of lymphocyte-derived cytokines from family members of persistent controllers were limited to increased IL-10 production in response to *C. albicans* hyphae and decreased IL-17 and IFNγ release upon exposure to phytohemagglutinin (PHA) and *M. tuberculosis*, respectively compared to family from non-controllers (Figures 5B and 5D; nominal *p* < 0.05). We did not detect significant differences in the absolute numbers of monocyte and lymphocyte counts comparing families of controllers and with families of non-controllers (Figure S2D). In line with the differences in trained immunity responses between HIV controllers and non-controllers, the exposure of both family groups to β-glucan resulted in an increased production of TNF, IL-6, and IL-1Ra upon LPS restimulation, compared to RPMI-exposed monocytes (Figure 5E, *p <* 0.05). Of note, the cytokine production of family members of persistent controllers was higher than that of family members of non-controllers both prior to and after β-glucan exposure; therefore, the fold increase of TNF, IL-6, and IL-1Ra was the same between both groups (Figure 5F). Lastly, we measured higher circulating β-glucan concentrations in the plasma of family members of persistent controllers than in the plasma of families of non-controllers. In addition, persistent controllers also had a tendency to higher β-glucan concentrations (*p* = 0.054) than non-controllers (Figure S2E). Together, our results demonstrate that family members of subjects with persistent HIV control status had significantly stronger innate and trained immune responses compared with the family members of non-controllers. This suggests a pre-existing favorable trained immunity phenotype in HIV controllers versus non-controllers.

## Discussion

Our study demonstrates that the presence of a HIV controller status, especially that of EC, is associated with more potent innate immune responses compared to a non-controller status of HIV normal progressors using ART. This conclusion is based on complementary immunological, genomic, epigenomic, and transcriptional datasets in cohorts of HIV controllers and non-controllers. Importantly, this conclusion is supported by an independent validation in cohorts of first-degree family members, showing that family members of PLHIV with HIV controller status have stronger innate immune responses compared to family members of non-controllers. Based on the data of immune responses of uninfected family members as surrogates of the immune responses of elite controllers prior to HIV infection, it can be proposed that elite controllers are likely to control HIV infection by a strong innate immune baseline. These data are reminiscent of earlier studies showing that parents of children who survived meningococcus sepsis have higher cytokine production capacity compared to parents of children who died of meningococcus sepsis,^26^ arguing that strong innate immune responses are crucial for the control of both bacterial and viral infections in the very first stages of pathogen invasion.

The assessment of cytokine production capacity of HIV controllers and their families showed a stronger release of cytokines produced by monocytes. In contrast, the production of proinflammatory cytokines produced by T cells, such as IFNγ, was higher in HIV controllers vs. non-controllers, but not in their relatives. This supports the concept that the strong T cell responses in the HIV controllers are likely secondary to the HIV infection, indicating that the adaptive immune system may play an important role in maintaining control status in the chronic phase, whereas monocyte-mediated innate responses appear to be crucial during the early stages of infection. At the transcriptional level, the cell type with the most DEGs between controllers and non-controllers was the classical monocytes. Notably, elite controllers showed significant enrichment for genes of the interferon pathway, a group of genes known for their effects against various viral infections, including HIV.^21^^,^^22^^,^^27^^,^^28^ For example, the guanylate binding protein (GBP) 5, highly expressed in our cohort of HIV controllers, is an interferon-inducible molecule, which inhibits HIV infectivity by impairing the viral assembly process in macrophages.^29^ Notably, the expression profile of the inflammatory genes, such as *IL-1B* and *CXCL8*, was lower in EC-monocytes, highlighting the importance of toning down inflammation to achieve HIV control. In line with our findings, the downregulation of innate immune pathways in viremic PLHIV compared to long-term non-progressors, including IRFs, was reported to be associated with disease progression in a previous study.^30^ The association between control of viral load and the epigenetic signature identified in ECs was confirmed in a cohort of ART-treated Rhesus Macaques (RMs) who received infusions of anti-PD-1 and anti-IL-10 (personal communication). These RMs were able to control viral load rebound to levels below 1000 copies of RNA per mL; moreover, a subset of these RMs showed a decay in cell-associated viral DNA (CA-vDNA). Flow cytometry, cytokine profiling, bulk RNA-seq, scRNAseq/scATAC seq all showed that the EC signature that included the open chromatin and transcriptional upregulation of the type I/Type II interferon pathway was a feature of RMs that controlled viral load rebound and showed a decay in frequencies of cells with vDNA.

GWAS studies have earlier demonstrated the involvement of genetic variants in the MHC region and *CCR5* locus in HIV infections, but they do not fully explain the HIV control status.^6^^,^^7^ In our study, HLA-B∗07:02 and HLA-C∗07:02 alleles associated with HIV control were found to significantly influence the CMV-induced cytokine production, implying a potential role of HLA alleles in shaping CMV-specific responses among HIV controllers. In addition to genetics, our findings argue for additional non-genetic immunological mechanisms. Long-term regulation of innate immune function can be induced by certain stimuli through epigenetic regulation, a process termed *trained immunity*.^23^ Importantly, both HIV controllers and their family members displayed a stronger capacity to induce trained immunity after β-glucan exposure. Although the source of circulating β-glucan was not investigated, this observation was strengthened by epigenetic changes at the level of chromatin marks associated with chromatin accessibility in immune genes of controllers. In a previous smaller-scale study (200-HIV), we reported that monocytes of PLHIV using long-term ART display a trained immunity phenotype characterized by increased β-glucan plasma concentrations and stronger IL-1β production capacity, two hallmarks of trained immunity.^31^^,^^32^ The observation from the present study suggests that different trained programs can be induced in PLHIV. On one hand, a more inflammatory trained immunity program can enhance the capacity to produce proinflammatory cytokines, while on the other hand, an anti-viral trained immunity program characterized by the transcription of interferon (IFN)-stimulated response elements (ISREs) and IRFs^33^ may reduce viral replication and promote resistance to HIV infection. Specifically, in HIV controllers, the persistence of interferon-inducible responses appears to be crucial for the maintenance of viral control. Underscoring the importance of the epigenetic regulation of innate immune cells in HIV control, a recent proof-of-principle study has demonstrated that the combination of the histone deacetylase inhibitor panobinostat with pegylated IFNα2a induced changes in the HIV reservoir similarly to those of ECs (HIV proviruses were integrated in genomic regions with reduced chromatin accessibility). This observation was aligned with phenotypic change in cells from the innate immune compartment, namely plasmacytoid and myeloid DCs, and a rise in monocytes and natural killer cells.^34^

In conclusion, our data suggest that monocytes are an additional important player in orchestrating the immune responses that are important for the establishment of spontaneous HIV control, and suggest that a trained immunity-like phenotype is the underlying mechanism. One limitation of our study is the fact that most of the persistent HIV controllers are viremic controllers receiving ART. Therefore, we cannot rule out the potential confounding effects of ART on innate immune responses in this specific subgroup of controllers. The ability of monocytes to mount effective responses upon encounter with a pathogen may also be beneficial in controlling the burden of coinfections in PLHIV.^35^ Importantly, our results open the door for designing new interventions, such as vaccines and adjuvants, which promote innate immune responses, aimed at replicating the responses identified in ECs. Indeed, the ASO3-adjuvanted H5N1 influenza vaccine was shown to induce similar responses in myeloid cells, achieving heterologous protection against other viruses.^36^ Future studies using vaccines and immunostimulants that induce antiviral trained immunity programs are warranted to assess their potential to induce HIV control in PLHIV.

### Limitations of the study

In this study, we demonstrated that enhanced monocyte functionality is a characteristic of spontaneous HIV controllers. Although we included first-degree relatives of both spontaneous controllers and non-controllers to show that a pre-existing favorable trained immunity phenotype may contribute to spontaneous control, our study in PLHIV did not allow us to distinguish whether the immune phenotype was already present before HIV infection or induced by the infection itself. Further studies combining trained immunity approaches with non-human models of HIV infection could provide additional insights into the contribution of monocyte responses to the development of a protective phenotype in HIV infections. Lastly, the group of controllers from our study is heterogeneous: there are viremic and non-viremic, persistent and transient controllers, as well as ART-naïve and ART-exposed controllers. The inclusion of a larger group of non-ART-treated elite controllers would have been preferable, however, as the current treatment protocols mandate that all PLHIV are given ART, which, in combination with the rarity of the elite controller phenotype, seriously limits the availability of such candidates. Future efforts on gathering archived PBMCs samples from previous longitudinal studies (pre- and post-ART) would be necessary to investigate the impact of ART on innate immune responses in persistent elite controllers.

## Resource availability

### Lead contact

Further information and requests for resources should be directed to and will be fulfilled by the lead contact, Mihai G. Netea (mihai.netea@radboudumc.nl).

### Materials availability

This study did not generate unique reagents.

### Data and code availability

• scRNA sequencing and ChIP sequencing have been deposited in the Radboud Data Repository (https://doi.org/10.34973/n2xg-3b05) and are publicly available as of the date of publication.
• This article does not report original code.
• Any additional data reported in this article will be shared by the lead contact upon request.
• Any additional information required to reanalyze the data reported in this article is available from the lead contact upon request.

## Acknowledgments

This study was supported by 10.13039/100010877ViiV Healthcare (A18-1052). We thank all volunteers who were willing to participate in this study. In addition, we thank Karin Grintjes, Manon Marneef-Pietersma, Safanatunnajah, Ayten Karisli, Fieke Weren, Liz Fransman, Pepijn van Kempen, Hanneke Maas, Malin Östman, and Jelmer van Puffelen for their help in recruiting participants and for their contributions in the lab. We thank Maria Guarini as well as the PRECISE platform at DZNE for the assistance with sample preparation, sequencing, and data generation.

## Author contributions

M.G.N. and A.J.A.M. vdV. conceptualized the study. J.C.dS. led, performed the analyses, and wrote the article. A.L.G. recruited the participants of the 2000HIV-trained study. S.D.E.R. performed the GWAS analyses. R.K. performed the analysis of sc-RNAseq. N.V. performed the plasma proteomics analysis. R.tH., S.R., C.B., and J.H.A.M. performed the epigenetic analysis. A.vdM., H.K., and M.I.dJ. generated the HLA next-generation sequencing. E.T.F. assisted with the ChIP-seq protocol and with the design of the figures. W.W. assisted with the optimization of protocols. M.J.T.B., L.E.vE., W.A.J.W.V., A.L.G., and T.O. recruited the participants of the 2000HIV study cohort. M.C.J. coordinated the logistics during the recruitment of the participants. J.vL., L.A.B.J., C.R., L.V., and A.V. assisted during the establishment of the cohort. M.D.B., A.C.A., and J.L.S. supervised sc-RNAseq work. V.M. performed the analysis of the MHC-genetics data and overall supervised the genetic analysis. J.C.dS assembled the figures. M.G.N. and A.J.A.M. vdV supervised the study. All authors critically revised the article for intellectual content. All authors read and approved the final version of the article.

## Declaration of interests

The authors are part of the 2000HIV study, which is supported by ViiV Healthcare. MGN is a scientific founder of TTxD, Biotrip, Lemba and Salvina.

## STAR★Methods

### Key resources table

**Table:** 

REAGENT or RESOURCE	SOURCE	IDENTIFIER
**Antibodies**		

Human Fc block	BD Pharmingen	Cat#564219

**Bacterial and virus strains**		

*Streptococcus pneumoniae*	ATCC	ATCC 49619
*Escherichia coli*	ATCC	ATCC 35218
*Mycobacterium tuberculosis,* H37Rv whole cell lysate	BEI Resources	Cat#NR-14822
*Staphylococcus aureus*	ATCC	ATCC 29213
*Candida albicans*, strain UC820	ATCC	ATCC MYA-3573

**Biological samples**		

Human Pool Serum	Internal	–

**Chemicals, peptides, and recombinant proteins**		

HIV envelope peptide pool	JPT Peptide Solutions	Cat#PM-HIV-ENV
pp65-cytomegalovirus (CMV) peptide pool	JPT Peptide Solutions	Cat#PM-PP65-2
Imiquimod	Invivogen	Cat#tlr-imq
Lipopolysaccharide	Sigma-Aldrich, further purified in-house	Cat#L4005
PolyI:C	Invivogen	Cat#tlr-pic-5
Phytohemagglutinin (PHA)	Merck	Cat#L9017
IL-1α	R&D Bio-techne	Cat#200-LA-010
β1,3-(D)-glucan	Prof. David Williams, College of Medicine, Johnson City, USA	–

**Critical commercial assays**		

Human IL-1 beta/IL-1F2 DuoSet ELISA	R&D Bio-techne	Cat#DY201
Human IL-1ra/IL-1F3 DuoSet ELISA	R&D Bio-techne	Cat#DY280
Human IL-6 DuoSet ELISA	R&D Bio-techne	Cat#DY206
Human IL-8/CXCL8 DuoSet ELISA	R&D Bio-techne	Cat#DY208
Human IL-10 Duoset ELISA	R&D Bio-techne	Cat#DY217B
Human CCL2/MCP-1 DuoSet ELISA	R&D Bio-techne	Cat#DY279
Human CCL3/MIP-1 alpha DuoSet ELISA	R&D Bio-techne	Cat#DY270
Human TNF-alpha Duoset ELISA	R&D Bio-techne	Cat#DY210
Human IL-5 DuoSet ELISA	R&D Bio-techne	Cat#DY205
Human IL-17 DuoSet ELISA	R&D Bio-techne	Cat#DY317
Human IL-22 DuoSet ELISA	R&D Bio-techne	Cat#DY287
Human IFN-gamma DuoSet ELISA	R&D Bio-techne	Cat#DY285B
BDG assay	Fungitell	Cat#FT001
CD14^+^ Microbeads	Miltenyi Biotec	Cat#130-097-052
Qubit dsDNA HS kit	Thermo-Fisher	Cat#Q32851
Abseq Immune Discovery panel	Becton Dickinson	Cat#625970
Human Single-Cell Multiplexing Kit	Becton Dickinson	Cat#633781
BD Rhapsody Cartridge Kit	Becton Dickinson	Cat#633733
BD Cartridge Reagent Kit	Becton Dickinson	Cat#633731
BD Rhapsody cDNA Kit	Becton Dickinson	Cat#633773
BD Rhapsody WTA Amplification Kit	Becton Dickinson	Cat#633801
AMPure XP magnetic beads	Beckman Coulter	Cat#A63880
NGSgo-MX11-3 kit	GenDx	Cat#7971864
NGSgo Library Full Kit	GenDx	Cat#2842256
Illumina Infinium iSelect 24x1 HTS Beadchip kit	Illumnia	Cat#WG-405-1014
CUT&RUN Assay kit	Cell Signaling	Cat#86652
KAPA HyperPrep Kit	Roche	Cat#KK8502
High Sensitivity DNA Kit for 2100 Bioanalyzer Systems	Agilent	Cat#5067
Olink® Explore 3072	Olink	https://olink.com/products/olink-explore-3072-384

**Deposited data**		

Raw and analyzed data	This paper	https://doi.org/10.34973/n2xg-3b05
Code	N/A	This paper does not report original code

**Software and algorithms**		

GraphPad Prism	Graphpad Software	https://www.graphpad.com
R statistical programming	N/A	RRID:SCR_001905
DESeq2	N/A	RRID:SCR_015687
Seurat pipeline (v4.3.0)	N/A	RRID:SCR_016341
the Gene Ontology (GO)	N/A	RRID:SCR_017505
Kyoto Encyclopedia of Genes And Genomes (KEGG)	N/A	RRID:SCR_012773
Reactome	N/A	RRID:SCR_003485
clusterProfiler version 4.0.5	N/A	RRID:SCR_016884
RcisTarget version 1.12.0	N/A	RRID:SCR_024860
PLINK v1.90b	N/A	RRID:SCR_001757
GRCh38	N/A	http://genome.ucsc.edu
TOPMed Freeze5	N/A	https://www.well.ox.ac.uk/∼wrayner/tools/)
NGSengine version 2.29.0	N/A	https://www.gendx.com/product_line/ngsengine/
MatrixEQTL	N/A	RRID:SCR_001757
HLA-TAPAS	Luo et al.^37^	https://github.com/immunogenomics/HLA-TAPAS
Picard	N/A	RRID:SCR_006525
ggplot2	N/A	RRID:SCR_014601

**Other**		

XN-1000 Automated Hematology Analyzer	Sysmex	XN-1000-010

### Experimental model and study participant details

#### Participants

We enrolled 1895 PLHIV from October 2019 until October 2021 as part of the 2000HIV study.^38^ The 2000HIV study is composed of discovery and validation cohorts of which the participants of the discovery cohort were recruited from three specialized Dutch HIV treatment centers, two university medical centers and one large general hospital (Radboudumc Nijmegen, Erasmus MC Rotterdam, and OLVG Amsterdam). Participants in the validation cohort were recruited in a separate specialized HIV center, a large general hospital (Elisabeth-TweeSteden Ziekenhuis Tilburg). The study population reflected a European HIV cohort, comprising 288 females (15.2%), 463 individuals of non-European ethnicity (24.4%), and 1360 men who have sex with men (MSM, 71.8%). The criteria for inclusion of the participants were HIV-1 infection, age of 18 years or older, receiving ART for at least six months and with a latest HIV-1 RNA load of less than 200 copies/mL. Besides 1721 PLHIV on ART, we enrolled 114 spontaneous HIV-1 controllers defined as i. non-viremic (elite) controllers (EC) characterized by HIV-1 RNA <75 copies/mL for more than 12 months in the absence of cART with stable CD4 T cell counts (>500 cells/mm^3^), ii. viremic controllers (VC) characterized by HIV-1 RNA <10.000 copies/mL for at least 5 years in the absence of cART with stable CD4 T cell counts and iii. transient controllers (Transient controllers) exhibited plasma HIV-RNA levels exceeding 10,000 copies/mL after initially meeting the criteria for being classified as an HIV controller. In contrast, persistent HIV controllers or persistent EC are individuals who managed to maintain their HIV-RNA levels less than 10,000 or 75 copies/mL respectively. ART was initiated in a subset of HIV controllers during follow-up due to factors, such as transmission prevention, patient preference or adherence to new HIV treatment guidelines introduced in 2015, which recommended ART for all PLHIV regardless of viral load levels or CD4 counts. The criteria for exclusion were absence of informed consent, insufficient communication because of language barriers or other problems, current pregnancy, detectable viral hepatitis B or C DNA as well as signs of any current acute infection. Spontaneous HIV-1 controllers included a higher proportion of females than normal progressors on ART in both the discovery (25.5%) and validation (33.3%) cohorts (Table 1). Sex differences between the PLHIV groups were accounted for in the statistical models, as described below. As for the 2000HIV-trained sub study, we enrolled PLHIV (persistent HIV-1 controllers and non-controllers on ART) and HIV-1 negative 1^st^ degree family members of HIV controllers and non-HIV controllers, respectively. Similar to the 2000HIV study, the majority of participants in the 2000HIV-trained sub study were of European ancestry, comprising of 43 females (45.3%) and 52 (54.7%) males. The proportions of females and males within the PLHIV groups were similar (Table 3). All participants were included between August and October 2021. For PLHIV, the inclusion criteria were the same as used in the 2000HIV study. The exclusion criteria for the family members were the presence of inflammatory comorbidities or use of immunomodulating medication usage and a positive serology for HIV-1. All participants were excluded in case of a recent infection or vaccination (<4 weeks) prior the date of inclusion. All study participants provided written informed consent. Due to careful (voluntary) registration of most PLHIV in the Netherlands in the ATHENA cohort (Stichting HIV Monitoring), a full overview of the general and HIV-related medical history of our participants was obtained.

#### Ethics

The 2000HIV and trained studies were approved by the Medical Ethical Review Committee Oost Nederland, Nijmegen, the Netherlands NL68056.091.81 and NL76999.091.21, respectively and published at clinicaltrials.gov (2000HIV study - NTC03994835 and 2000HIV Trained - NCT04968717).

### Method details

#### *Ex-vivo* stimulation of peripheral blood mononuclear cells (PBMCs)

Human peripheral blood mononuclear cells (PBMCs) were obtained from venous blood collected in EDTA tubes and were isolated density centrifugation of blood diluted 1:1 in pyrogen-free phosphate-buffered saline (PBS) over Ficoll-Paque (GE healthcare, UK). 500.000 PBMCs/well were seeded in U-bottom plates (Corning) and subsequently stimulated with bacterial (*Streptococcus pneumoniae, Escherichia coli*, *Mycobacterium tuberculosis, Staphylococcus aureus*), viral (HIV envelope pool and pp65-cytomegalovirus (CMV)), fungal (*Candida albicans conidia and hyphae*) and Toll-like receptor (TLR) ligands (imiquimod-IMQ, LPS and Poly:IC) for 24 h and 7 days at 37°C and 5% CO_2_. Table S4 contains the information of the supplier as well as the stimuli concentrations used in the experiments. The production of cytokines and chemokines (IL-1β, IL-1Ra, IL-6, IL-8, IL-10, MCP-1, MIP-1α, TNF, IL-5, IL-10, IL-17, IL-22 and IFN-γ) were assessed in the supernatants by ELISA (Duoset ELISA, R&D Systems).

#### Hemocytometry measurement

Hemocytometry was performed on whole blood with the Sysmex XN series hematology analyzer (XN) (Sysmex, Kobe, Japan) as described before.^38^

#### Plasma markers

IFN-γ and sCD14 markers were measured in EDTA-plasma samples using Olink Proteomics AB based on a proximity extension assay coupled with next-generation sequencing as readout method (Olink Explore panel). Statistical significance was assessed through linear regression adjusted for age and sex as confounders. β-glucan was assessed using the Fungitell BDG assay. Statistical significance was assessed through linear regression adjusted for age and sex as confounders.

#### BD Rhapsody single-cell RNA-sequencing

Frozen PBMCs were recovered by rapidly thawing frozen cell suspensions in a 37°C water bath followed by immediate serial dilution in pre-warmed RPMI1640 + 10% FBS (GIBCO) and centrifugation at 300*g* for 5 min. After centrifugation, the cells were resuspended in RPMI1640 + 10% FBS and processed for whole transcriptome analyses, using the BD Rhapsody Single-Cell Analysis System (BD, Biosciences) as previously described.^39^ Cells from each sample were labeled with hashtag-oligonucleotide-coupled antibodies, sample tags (BD Human Single-Cell Multiplexing Kit) following the manufacturer’s protocol. Briefly, a total number of 1 × 10^6^ cells were resuspended in 90 μL of Stain Buffer (FBS) (BD PharMingen). The sample tags were added to the respective samples and incubated for 20 min at room temperature. After incubation, 500 μL stain buffer was added to each sample and centrifuged for 5 min at 300 g and 4°C. Samples were washed one more time. Subsequently cells were resuspended in 300 μL of cold BD Sample Buffer and counted using Improved Neubauer Hemocytometer (INCYTO). Labeled samples were pooled equally in 650 μL cold BD Sample Buffer. Cells were then stained with antibodies using the BD AbSeq Immune Discovery Panel following the manufacturer’s protocol. In brief, pooled samples were centrifuged for 5 min at 300*g* and 4°C, supernatant was removed, 100 μL blocking buffer (Fc block) was added to the cell mixture and incubated for 10 min at RT. Cell suspensions were mixed with the antibody-solution and incubated for 40 min on ice. After incubation, 2 mL BD Stain Buffer was added and centrifuged for 5 min at 300*g* and 4°C. The cell pellet was resuspended in 300 μL Sample Buffer and cell were counted. For each pooled sample two BD Rhapsody cartridges were super-loaded with approximately 60,000 cells each. Single cells were isolated using Single-Cell Capture and cDNA Synthesis with the BD Rhapsody Express Single-Cell Analysis System according to the manufacturer’s recommendations (BD Biosciences). cDNA libraries were prepared using the BD Rhapsody Whole Transcriptome Analysis Amplification Kit following the BD Rhapsody System mRNA Whole Transcriptome Analysis (WTA), Sample Tag Library Preparation Protocol (BD Biosciences) and AbSeq Library Preparation Protocol (BD Biosciences). The final libraries were quantified using a Qubit Fluorometer with the Qubit dsDNA HS Kit (ThermoFisher) and the size-distribution was measured using the Agilent high sensitivity D5000 assay on a TapeStation 4200 system (Agilent technologies). Sequencing was performed in paired-end mode (2∗75 cycles) on a NovaSeq 6000 with NovaSeq 6000 S2 or S4 Reagent Kit v1.5 (200 cycles) chemistry.

#### Data pre-processing of scRNA-seq data

After demultiplexing of bcl files using Bcl2fastq2 V2.20 from Illumina and quality control, paired-end scRNA-seq reads were filtered for valid cell barcodes using the barcode whitelist provided by BD. Cutadapt 1.16 was then used to trim NexteraPE-PE adaptor sequences where needed and to filter reads for a PHRED score of 20 or above. Then, STAR 2.7.3a was used for alignment against the Gencode v27 reference genome.^40^ Dropseq-tools 2.0.0 were used to quantify gene expression and collapse to UMI count data (https://github.com/broadinstitute/Drop-seq/). For hashtag-oligo based demultiplexing of single-cell transcriptomes and subsequent assignment of cell barcodes to their sample of origin the respective multiplexing tag sequences as well as AbSeq sequences were added to the reference genome and quantified as well.

#### Single-cell RNA-seq data quality control and cell annotation

Analysis of scRNA-seq data was performed using the Seurat pipeline (v4.3.0).^41^^,^^42^ During preprocessing and quality control (QC), cells that were considered as doublets or negatives after genetic demultiplexing using vireo and linking to sample tags using the HTODemux function from Seurat (positive.quantile 0.99), singlets that did not exceed 500 unique molecular identifiers (UMIs), had more than 35% mitochondrial genes, showed less than 250 and more than 2500 features per cell or were present in small contaminating clusters were excluded from downstream analysis. Additionally, genes that were expressed in less than 5 cells per cartridge were removed. After QC, a total of 173,989 single-cell transcriptomes of PBMCs were analyzed. The RNA data of the entire dataset was normalized, scaled and dimensional reduction was calculated using the standard Seurat functions. For normalization, the gene expression values were normalized by total UMI counts per cell, multiplied by 10,000 (TP10K) and then log transformed by log10(TP10k+1). Subsequently, the data was scaled and centered. For dimensionality reduction, PCA was performed on the top 2,000 variable genes identified using the vst method. AbSeq data was normalized per cartridge using the dsb normalization including background correction.^43^ To combine both RNA and AbSeq information, a weighted nearest neighbor (WNN) analysis was performed as described in the Seurat pipeline.^41^ As dimensionality reductions, the first 20 principal components (PCs) were used for the RNA modality and all normalized antibody counts were used directly as an input for the antibody modality. For two-dimensional representation of the data structure, uniform manifold approximation and projection (UMAP) was calculated on the multi-modal WNN. Subsequently, the cells were clustered using the SLM algorithm with a resolution of 0.3. Cluster-specific marker genes were calculated with the Wilcoxon rank-sum test using the FindAllMakers function (min.pct = 0.2, logfc.threshold = 0.5). Using the combined information of cluster marker and literature-known markers, present cell types were annotated as described in Figure S1B.

#### Selection and annotation of monocytes (scRNA-seq)

Monocytes were selected and annotated in a four-step process. First, classical monocyte transcriptomes were subset from the PBMC data. This subset was subsequently normalized, scaled and dimensional reduction was calculated using the standard Seurat functions. For normalization, the gene expression values were normalized by total UMI counts per cell, multiplied by 10,000 (TP10K) and then log transformed by log10(TP10k+1). Subsequently, the data was scaled and centered. For dimensionality reduction, PCA was performed on the top 2000 variable genes identified using the vst method. For two-dimensional representation of the data structure, UMAP was calculated using the first 15 PCs. Next, sex-specific clusters were cleaned from monocytes. Finally, clusters were calculated using a resolution of 0.2, respectively. Cluster-specific marker genes were calculated with the Wilcoxon rank-sum test using the FindAllMakers function (min.pct = 0.2, logfc.threshold = 0.25).

#### Differential gene expression analysis (scRNA-seq)

Differential expression (DE) tests were performed using FindMarkers function from Seurat with the Wilcoxon rank-sum test. Genes with an absolute log2-fold change ≥0.25, at least 10% expressed in tested groups and with a bonferroni-corrected *p*-value ≤0.05 were considered as significantly differentially expressed genes (DEGs). To evaluate the robustness of classical monocyte DEG, their significance was assessed through 1,000 permutations. In each permutation, the EC group (*n* = 7) was held constant while an equally sized group of non-HIC (*n* = 7) was randomly selected for comparison. A robust DEG call was defined by an adjusted *p*-value below 0.05 in the majority of comparisons.

#### Quantification of percentages of cell types and states

To compare shifts in the PBMC cell types and monocyte states stratified by clinical groups, the percentages of each cluster were quantified per sample of the respective groups and visualized together in boxplots. Wilcoxon test was used for determination of statistically significant differences in the distribution.

#### Enrichment analyses

Functional enrichment analysis was performed using the monocyte DEG as input based on the gene sets from the Gene Ontology (GO) biological processes (BP) database^44^^,^^45^ and the Kyoto Encyclopedia of Genes And Genomes (KEGG) database,^46^ as well as the Hallmark^47^ and the Reactome^48^^,^^49^ gene sets using the R package clusterProfiler (version 4.0.5).^50^^,^^51^ Only terms with statistical significance (bonferroni-corrected *p*-value≤0.05) were considered. Transcription factor motif enrichment was performed with the R package RcisTarget (version 1.12.0).^52^ The genomic regions of TF-motif search were limited to 10 kb around the respective transcriptional start sites by using the RcisTarget-implemented “hg19-tss-centered-10kb-7species.mc9nr.feather” motifRanking file.

#### Genotyping, pre-imputation quality control and imputation

DNA was isolated from whole blood for all participants of multiple ancestries. DNA was genotyped on the Illumina Infinium Global Screening Array. Prior to imputation, quality control for raw variants and samples was performed. Quality control (QC) for raw variants and samples was performed using PLINK v1.90b.^53^ No sample was initially excluded due to failure in sex check (using sex-check from PLINK). Genetic variants from autosomes with a call rate genotype missingness of >5% and those deviating from Hardy-Weinberg equilibrium (HWE) with a *p* value <10^−6^ per ancestry were excluded from the dataset. HWE exact test was performed using variants stratified by ancestry. Samples with a call rate of <97.5% and those that showed a heterozygosity rate that deviated three standard deviations (SD) from the mean heterozygosity rate were excluded. Heterozygosity rate was calculated per ancestry. Genetic variants that passed QC were lifted from GRCh37 to GRCh38 build using the UCSC liftOver tool (http://genome.ucsc.edu).^54^ Next, strand alignment to the TOPMed reference panel (on GRCh38 build) was performed using the TOPMed Freeze5 on genome build GRCh38. For this alignment, we used the code providing with the McCarthy group tools (https://www.well.ox.ac.uk/∼wrayner/tools/). After QC, 582,404 variants from 1864 individuals from all ancestries were retained for the imputation procedure. The filtered raw variants were uploaded to the TOPmed Imputation server and imputed against the TOPMed (version r2 on GRCh38) reference panel. The imputed variants were filtered using BCF tools,^55^ excluding variants with low imputation quality scores (R2 < 0.3 or ER2 < 0.7) and MAF <1%. The variants were assigned reference SNP ID (rs) by BCFtools against variants (b151 GRCh38p7) downloaded from dbSNP. This resulted in 10,810,841 variants from 1864 individuals of the 2000HIV multi-ancestry cohort.

#### Post-imputation quality control

Since population stratification is known to be an important confounder in genome-wide association studies (GWASs), we performed a GWAS using individuals from only European ancestry. First, post-imputation QC was performed. Briefly, genetic variants with a MAF <0.05, deviating from HWE (*p* < 10^−12^), insertions, deletions and copy number variants were removed. Additionally, individuals with outlying heterozygosity (those that deviate three SD from the mean heterozygosity rate), discordant sex information (X chromosomal inbreeding coefficient >0.2 for females or <0.8 for men), with one sample identified as having klinefelter syndrome, as well as related individuals (based on the identity-by-descent, IBD >0.1875) were excluded. Ancestral outliers were removed, as defined by a distance of over 3 SD from the mean of the 1000 genomes European reference population on PC1 and PC2. After QC, 5,791,803 variants from 1271 individuals of European ancestry were retained. Finally, individuals with an unknown HIV controller status were excluded, leaving 1246 individuals for the GWAS on HIV control. Post-imputation quality control for variants and samples, and association testing HIV control was performed using PLINK v1.90b.^53^

#### GWAS on HIV control

We tested which imputed SNP variants were associated with HIV control using 67 HIV controllers and 1179 non-controllers. An additive logistic regression model was used in which age, sex and the first five genetic PCs, to correct for population stratification, were included as confounders (Figure S2A). SNPs with a *p* < 1 × 10^−^^5^ were considered to show a suggestive association with HIV control. Association testing was performed using PLINK v1.90b.

#### MHC imputation

To gain further insight and reveal extensive association within the MHC region, the Michigan imputation server was used to impute SNPs, alleles and amino acids in the MHC region (6:27,970,031–33,965,553) on GRCh37 build using the four-digit multi-ethnic HLA v2 reference panel.^37^ After the standard QC per SNP and sample as described above, genotyped variants from chromosome 6 from 1864 individuals of all ethnicities from the 2000HIV cohort were submitted for imputation. Genetic variants were lifted from GRCh38 to GRCh37 using the Michigan server. After imputation, we obtained 22,733 SNPs, alleles and amino acids within the MHC region, of which 570 were HLA classical alleles at two and four-digit resolution (HLA-A, HLA-B, HLA-C, HLA-DQA1, HLA-DQB1, HLA-DPA1, HLA-DPB1, HLA-DRB1) and 3449 amino acids, 4023 SNPs within HLA and 14691 scaffold SNPs. HLA alleles that showed a minor allele count (MAC) < 10 (*n* = 2826) and those at two-digit resolution were removed from follow-up analysis, resulting in a total of 19774 variants. Finally, to assess the accuracy of the HLA imputation, we tested the concordance between the sequence-based and imputed HLA alleles using 104 samples. For this, we calculated the percentage of samples for which the same HLA allele at four-digit resolution was present in both typed and imputed data taking into account homozygosity or heterozygosity status. A total of 157 HLA alleles were tested for concordance in 104 samples with both typed and imputed data. For follow up analysis, we focused on the four-digit resolution HLA alleles and amino acids within the MHC region.

#### Next generation sequencing of HLA alleles

HLA class I (HLA-A, -B, -C) and class II (HLA-DPA1, -DPB1,-DQA1, -DQB1, -DRB1, -DRB3, -DRB4, -DRB5) genes, were amplified by long-range PCR using the NGSgo-MX11-3 kit (GenDX, Utrecht, The Netherlands), to generate HLA amplicons, following the manufacturer’s instructions. HLA libraries were prepared using the NGSgo Library Full Kit following the manufacturer’s instructions. Paired-end sequencing (read length 2× 150 bp) was performed on the iSeq 100 System, Illumina. The fastq files generated and exported from the iSeq 100 System, Illumina, were analyzed using the analysis software NGSengine, (version 2.29.0, HLA IMGT database 3.51.0).

#### Association regression analysis using the HLA alleles

To test for association between HLA SNPs, alleles, amino acids, and HIV control, a firth logistics regression model with age, sex and the first five genetic PCs to account for population stratification, were used as covariates using the HLA-TAPAS pipeline.^37^ For this analysis, 67 HIV controllers and 1179 controls of European ancestry were used. The number of PCs were selected based on the PCA analysis using the GWAS data of the European cohort. A *p* value after multiple testing correction <2.53 × 10^−6^ (0.05/19.774) was considered to call significant associations, where 19774 is the total number of MHC-imputed SNPs, alleles, and amino acids tested. None of the HLA alleles tested reached statistical significance. In addition, an omnibus test is performed to determine the associate between each amino acid position and HIV control, accounting for all *m* amino acid residues changes occurring at that position as implemented in HLA-TAPAS pipeline.^37^

Lastly, to test for association between HLA alleles and *ex vivo* cytokine production, we used two independent cohorts of PLHIV, a discovery and validation cohort, both of European ancestry. Both MHC imputed data and *ex vivo* cytokine data upon 24-h stimulation were obtained for a total of 951 and 247 samples from the discovery and validation cohort, respectively. For 7-day stimulations, 958 and 245 samples were obtained from the discovery and validation cohort, respectively. In particular, 19533 MHC variants with MAC >10 in the discovery cohort were tested for association with *ex vivo* cytokine data. To test for association, a linear regression model was used with age, sex, seasonality, and COVID-19 vaccination as covariates. A *p* value after multiple testing correction <1.13 × 10^−4^ (0.05/19.533 × 44 pairs of stimuli) and <8.45 × 10^−5^ (0.05/19.533 × 33 pairs of stimuli) was considered to call significant associations for both 24 h and 7 days cytokines, respectively. Associating testing between HLA alleles and *ex vivo* cytokine data was performed using the MatrixEQTL package^56^ in R version 4.1.2.

#### Trained immunity induction in adherent monocytes

Adherent monocytes were trained as described previously by.^57^ Briefly, 500.000 cells were incubated either with culture medium containing 10% pooled human serum, referred to as complete medium, as a negative control, 1 μg/mL of β-glucan (β-1,3-(D)-glucan (kindly provided by Professor David Williams, College of Medicine, Johnson City, USA). After 24 h (37°C), cells were washed three times with 200 μL of warm PBS and incubated for 5 days with one change of complete medium. On day 6, cells were restimulated with either 200 μL RPMI, 10 ng/mL of lipopolysaccharide (LPS) derived from *E. coli* serotype O55:B5 (LPS - Sigma) for 24 h.

#### Cytokine measurements upon training

Cytokine production was determined in supernatants using commercial ELISA kits (R&D Systems) for human TNF, IL-6, IL-1Ra.

#### ChIP sequencing of CD14^+^ monocytes

CD14^+^ monocytes from persistent HIV controllers and non-controllers of the 2000HIV-trained study were enriched using MACS kit (CD14 Microbeads human, positive selection, Miltenyi Biotec), according to the manufacturer’s protocol. The yield was assessed on the Sysmex and purity of the CD14^+^ cells using flow cytometry. The median purity yield of the monocyte isolation was 75%. H3K4me3 chromatin enrichment was assessed in 200.000 CD14^+^ monocytes through CUT&RUN method^58^ according to the manufacturer’s recommendations (Cell Signaling). ChIP-seq libraries were prepared using the Kapa Hyper Prep Kit according to manufacturer’s protocol, with the following modifications. 2.5 mL of the NEXTflex adaptor stock (600 nM, Bioo Scientific) was used for adaptor ligation of each sample. Libraries were amplified with 12–15 PCR cycles followed by a double post-amplification clean-up was used to ensure proper removal of adapters. Samples were analyzed for purity using a High Sensitivity DNA Chip on a Bioanalyzer 2100 system (Agilent). Libraries were paired-end sequenced to a read length of 50 bp on an Illumina NextSeq500. ChIP-sequencing data was analyzed using Seq2science.^59^Briefly, raw sequencing reads were aligned to human genome hg38 with BWA.^60^ Samtools was used to filter reads with a quality score lower than 20, and PCR duplicates were removed with Picard.^61^ Peaks were identified with MACS 2.2.6 in paired-end mode and ‘call-summits’ enabled at a false discovery rate of 0.01.^62^ A union of all identified peaks was generated with BEDTools, which was used to count reads per peak in each sample.^63^ Significantly altered peaks were filtered using the following settings: Sum of average reads per peak >50 and fold difference of mean ± 2x stdev. Heatmaps were based on row normalized z-scores and generated using Fluff.^64^ Heatmap was generated based on row normalized z-scores of significantly altered peaks and generated using ggplot2 R package. Pathway enrichment analysis was conducted against the Hallmark gene sets^65^ using the clusterProfiler R package, with the closest gene to significantly altered peaks serving as input.

### Quantification and statistical analysis

#### Statistical analysis of cytokines

To test for differences in cytokines, chemokines and plasma proteins across the different PLHIV groups, we used a rank-based (rfit) regression model with age, sex, ethnicity and seasonality as confounders. Seasonality was modeled using a sine and cosine wave with a period of 365.25 days as previously described.^66^ Together, these two terms can form a sine wave with any phase with a frequency of a year. To account for ancestry, we calculated the first 5 genetic PCs using the genome-wide imputed genetic data of Europeans (discovery cohort) and included them in our model. To identify potential confounders, we assessed associations between the first five PCs as calculated using the data of interest (cytokines, chemokines or plasma proteins) and potential confounders using a linear regression model in the discovery cohort. We selected as potential confounders those that showed a beta coefficient >0.04 from a linear regression.

In particular, we compared (i) 95 HIV controllers versus 1317 non-HIV controllers, (ii) 52 persistent controllers versus 1317 non-HIV controllers, (iii) 43 transient controllers versus 1317 non-HIV controllers and (iv) 20 elite controller vs. 1317 non-HIV controllers.

To account for the imbalance in sample size between elite controllers and non-controllers, bootstrapping was performed keeping the group of HIV controllers consistent and randomly sampling the non-controllers using the sample function from R, with the replace parameter set to TRUE, using 500 iterations. For each iteration, a rfit model correcting for age, sex, seasonality and five genetic PCs was run, with scores parameter set to bentscores1, analogous to the original testing procedure. We considered the comparisons robust if the distribution of *p* values from all iterations falls within the 95% confidence intervals (Figures 3 and 4).

For determination of statistical significance in cytokines and chemokines between the family members groups, we used Wilcoxon rank-sum test.

### Additional resources

This work is not part of or involving a clinical trial.
