# Supplementary material for: Persistent viral control status is associated with enhanced innate immune responses in people with HIV-1
Source: iScience. 2026 Jan 30;29(3):114807. doi: 10.1016/j.isci.2026.114807 (PMC12973709; doi:10.1016/j.isci.2026.114807)
Supplement: Document S1. Figures S1–S4 [file mmc1.pdf]

iScience, Volume *29*

Supplemental information

Persistent viral control status

is associated with enhanced innate

immune responses in people with HIV-1

Jéssica C. dos Santos, Albert L. Groenendijk, Suzanne D.E. Ruijten, Rainer Knoll, Nadira Vadaq, Rob ter Horst, Ezio T. Fok, Wojciech Witkowski, Marc J.T. Blaauw, Louise E. van Eekeren, Wilhelm A.J. W. Vos, Maartje Cleophas-Jacobs, Stephan Reichl, Twan Otten, Joost H.A. Martens, Arnold van der Meer, Han Koninkx, Marien I. de Jonge, Marc D. Beyer, Jan van Lunzen, Leo A.B. Joosten, Christoph Bock, Casper Rokx, Annelies Verbon, Linos Vandekerckhove, Anna C. Aschenbrenner, Joachim L. Schultze, Vasiliki Matzaraki, Andre J.A.M. van der Ven, and Mihai G. Netea

Supplemental information

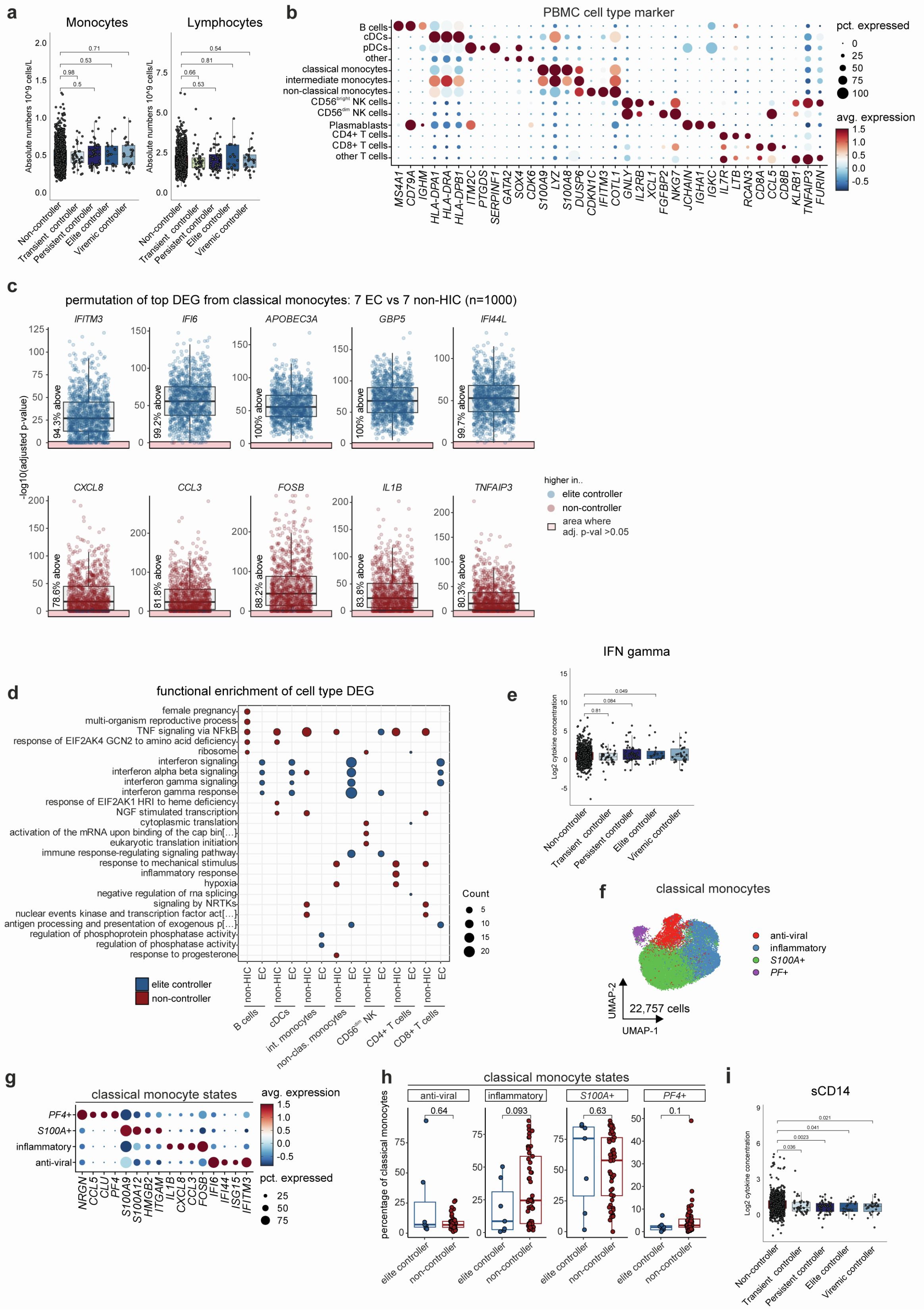

Figure S1 – Absolute cell counts, scRNA-seq and protein measurements, related to Figures 1-2

(A) Absolute counts of monocytes and lymphocytes assessed in whole blood of EC, VC, TC and non-controllers with the XN-1000 hematology analyzer. The data is represented in boxplots showing the median with standard errors. For determination of statistical significant differences in the distribution a Wilcoxon rank sum test was performed ($P < 0.05$). (B) Dot plot of cluster marker expression characterizing PBMCs of persistent controllers and non-controllers using scRNA-seq. (C) Permutation (n=1,000) of DEG calling to test robustness of selected identified monocyte DEG. For each permutation, ECs (n=7) were compared to randomly selected non-controllers (n=7) and the resulting adjusted p-value reported (Red area indicates tests with non-significant differences). (D) Functional enrichment for EC-specific up- and down-regulated genes in other cell major cell types, EC and non-controllers. (E) Plasma IFNγ levels of transient, persistent controllers EC, VC and non-controllers assessed with Olink. ($P < 0.05$; linear regression model with age and sex as covariates). (F) UMAP of classical monocytes with annotated states. (G) Top marker of classical monocytes. (H) Monocyte cluster distribution (represented as percentages) between EC and non-controllers. For determination of statistically significant differences in the distribution, a Wilcoxon rank sum test was performed. (I) Plasma CD14 levels of transient, persistent controllers EC, VC and non-controllers assessed with Olink. ($P < 0.05$; linear regression model with age and sex as covariates).

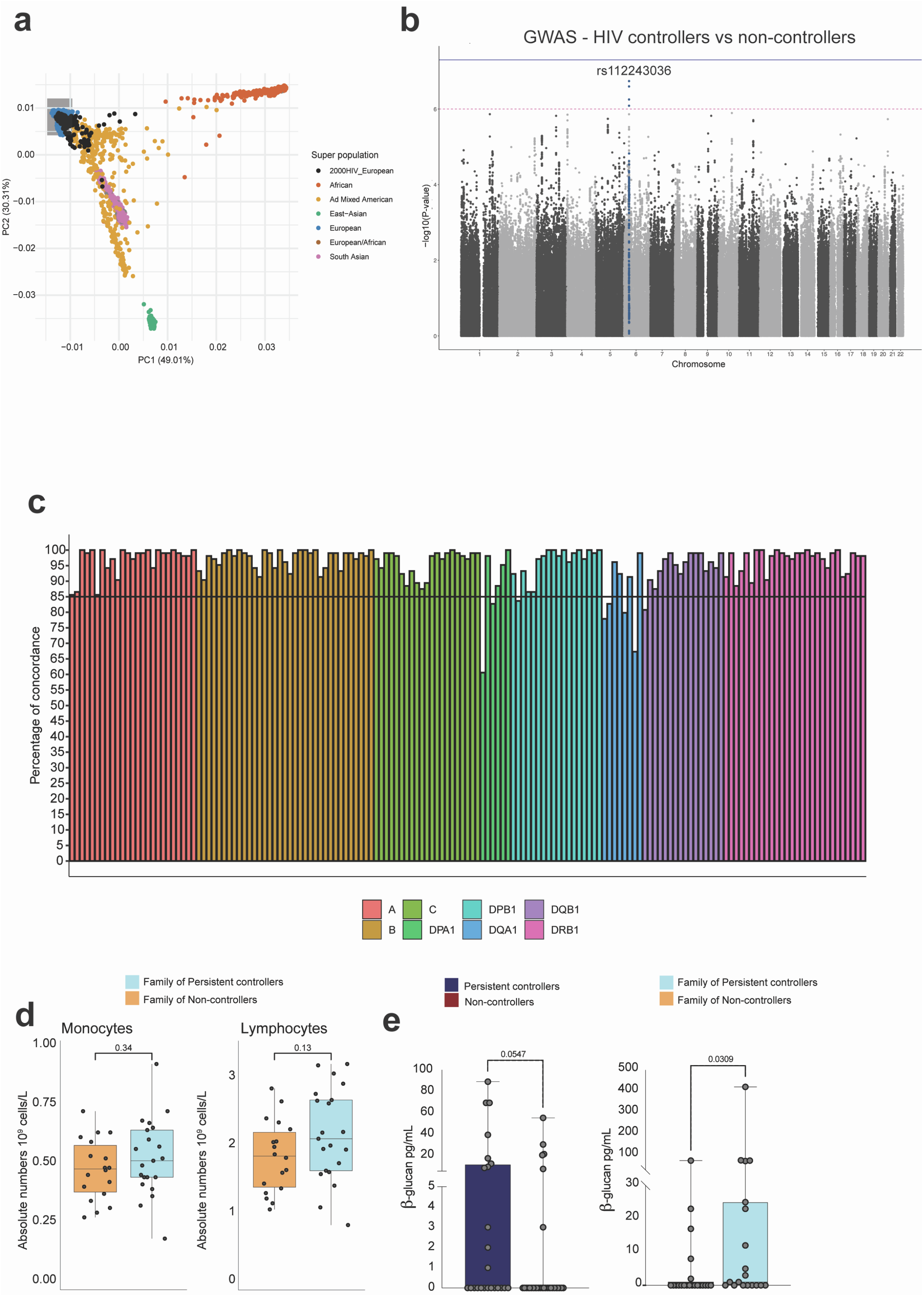

Figure S2 - GWAS, HLA typing, absolute counts and β-glucan levels, related to Figures 3-5

(A) Principal component analysis (PCA) showing the first two principal components for individuals of the 2000HIV cohort (HIV controllers and non-controllers) together with

individuals from the 1000genomes project. Each dot represents an individual. (B) Manhattan plot of genome-wide association study for HIV control, showing the -log10(*P*-value) (y-axis) against the chromosomal position (x-axis). The dotted line represents the threshold for suggestive association ($P < 1 \times 10^{-6}$). (C) HLA typing concordance plot showing the concordance of MHC imputation from genetic data and MHC typing using next generation sequencing. (D) Absolute counts of monocytes and lymphocytes assessed in whole blood of family members of persistent controllers and family of non-controllers with the XN-1000 hematology analyzer. (E) β-glucan concentrations in the plasma of 1st degree family members and PLHIV part of the 2000HIV-trained study.

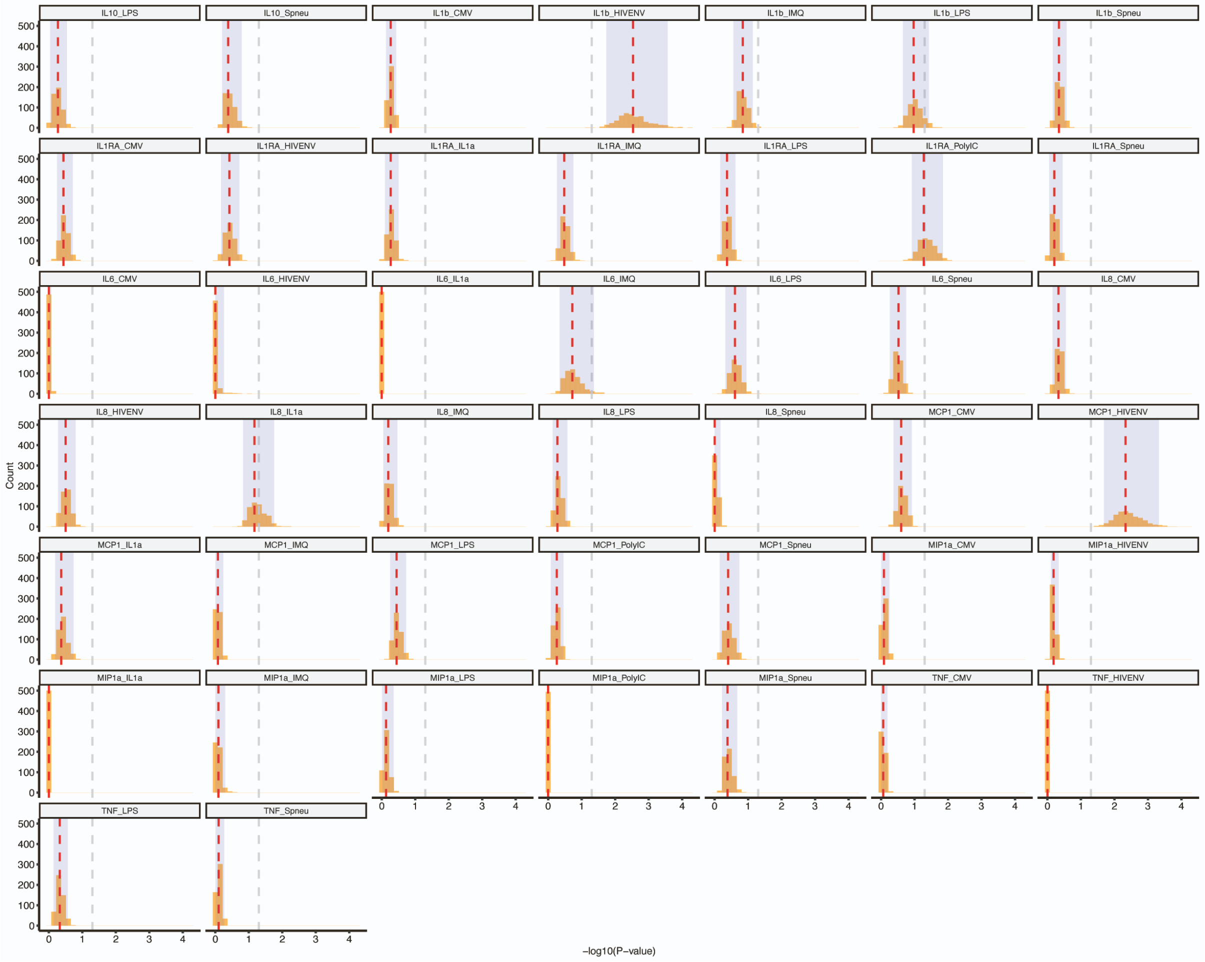

Figure S3 – Bootstrapping, related to Figure 1

Results from resampling of all EC versus non-controllers comparisons of stimuli pairs used on PBMCs for the 24 hours. Red dashed line indicates P value < 0.05. The shaded area indicates 95% confidence interval.

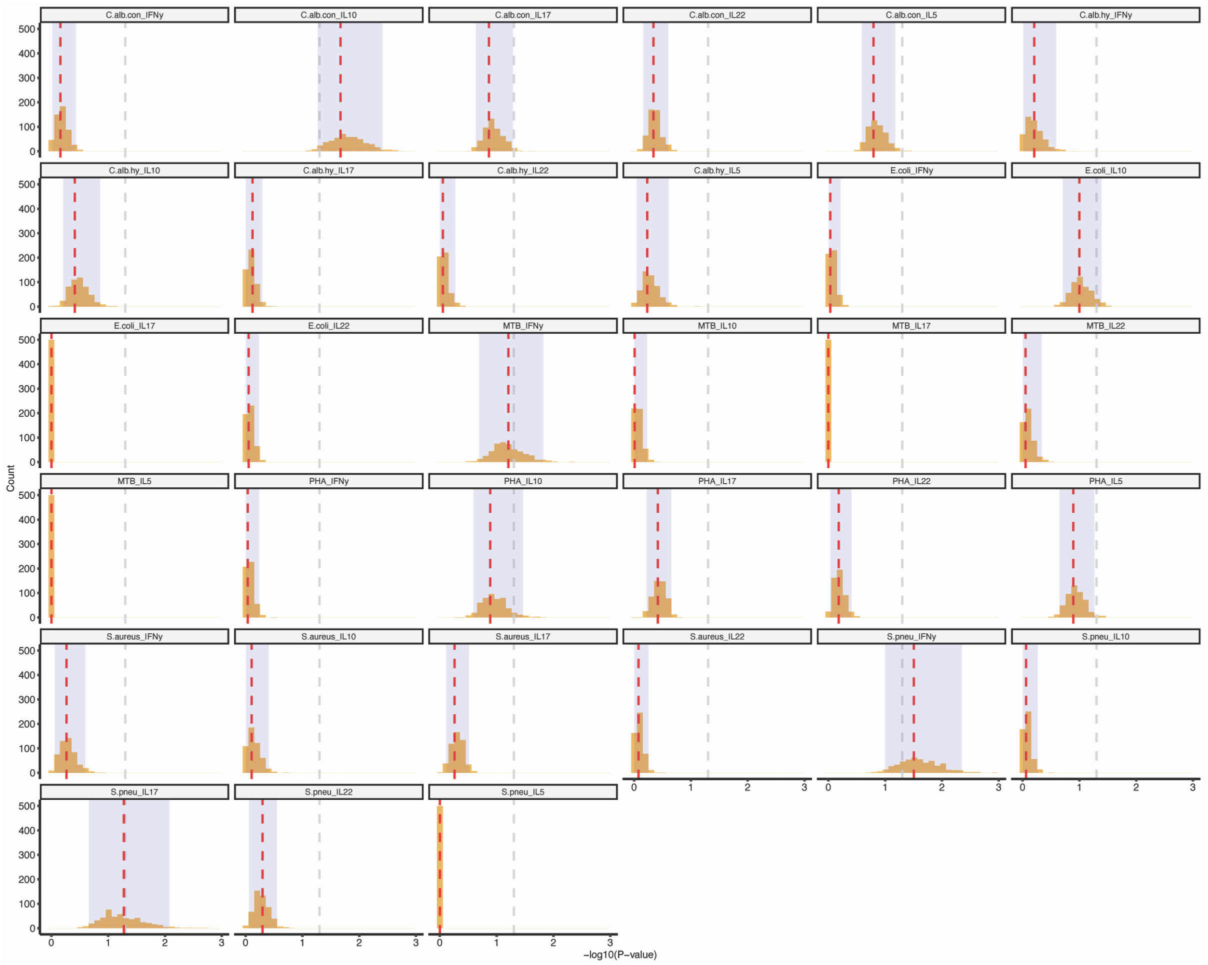

Figure S4 – Bootstrapping, related to Figure 1

Results from resampling of all EC versus non-controllers comparisons of stimuli pairs used on PBMCs for the 7 days. Red dashed line indicates P value < 0.05. The shaded area indicates 95% confidence interval.
